# Structural basis for non-canonical integrin engagement by *Bordetella* adenylate cyclase toxin

**DOI:** 10.1016/j.celrep.2022.111196

**Published:** 2022-08-16

**Authors:** Jory A. Goldsmith, Andrea M. DiVenere, Jennifer A. Maynard, Jason S. McLellan

**Affiliations:** 1 Department of Molecular Biosciences, The University of Texas at Austin, Austin, TX 78712, USA; 2 Department of Chemical Engineering, The University of Texas at Austin, Austin, TX 78712, USA; 3 Lead contact

## Abstract

Integrins are ubiquitous cell-surface heterodimers that are exploited by pathogens and toxins, including leukotoxins that target β_2_ integrins on phagocytes. The *Bordetella* adenylate cyclase toxin (ACT) uses the α_M_β_2_ integrin as a receptor, but the structural basis for integrin binding and neutralization by antibodies is poorly understood. Here, we use cryoelectron microscopy to determine a 2.7 Å resolution structure of an ACT fragment bound to α_M_β_2_. This structure reveals that ACT interacts with the headpiece and calf-2 of the α_M_ subunit in a non-canonical manner specific to bent, inactive α_M_β_2_. Neutralizing antibody epitopes map to ACT residues involved in α_M_ binding, providing the basis for antibody-mediated attachment inhibition. Furthermore, binding to α_M_β_2_ positions the essential ACT acylation sites, which are conserved among toxins exported by type I secretion systems, at the cell membrane. These findings reveal a structural mechanism for integrin-mediated attachment and explain antibody-mediated neutralization of ACT intoxication.

## In brief

*Bordetella* adenylate cyclase toxin (ACT) targets leukocytes via surface-expressed integrin α_M_β_2_. Goldsmith et al. use cryoelectron microscopy to reveal the structural basis for this non-canonical interaction and show that receptor binding is physically coupled to ACT membrane insertion. This study provides mechanistic insight into ACT function and its integrin-mediated attachment.

## Graphical Abstract

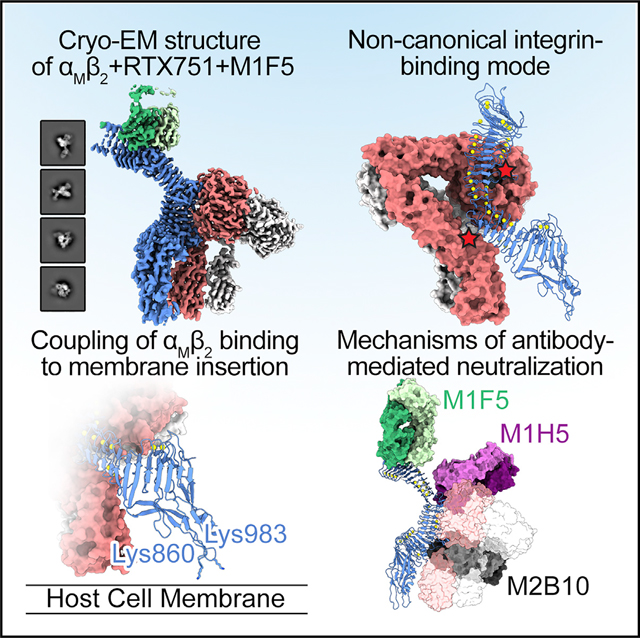

## INTRODUCTION

Integrins are a family of cell-surface heterodimers that, in animals, mediate cell-cell and cell-extracellular matrix adhesion and serve as receptors for a variety of ligands. Each integrin is made up of one α subunit and one β subunit, and the canonical ligand-binding metal-ion-dependent adhesion site (MIDAS) (Lee et al., 1995b) is within the N-terminal “headpiece.” Integrin activation involves large-scale conformational changes that are coupled to a reorganization of the ligand-binding site (Emsley et al., 2000; Lee et al., 1995a, 1995b; Shimaoka et al., 2003; Takagi et al., 2002). For many integrins, the inactive conformation is the “bent” state, where the integrin headpiece points toward the membrane at an approximately 20° angle relative to the membrane-anchored “tailpiece” (Nishida et al., 2006; Takagi et al., 2002, 2003; Xiong et al., 2001). During activation, a reorganization of the MIDAS allows for high-affinity ligand binding (Emsley et al., 2000; Lee et al., 1995a, 1995b; Shimaoka et al., 2003; Takagi et al., 2002), which is coupled to global conformational changes such as a large hinge motion of the headpiece into the “extended” conformation, as well as separation of the α and β subunit legs and cytoplasmic domains (Nishida et al., 2006; Takagi et al., 2002). Separation of the cytoplasmic domains connects the headpiece state to intracellular signaling (Kim et al., 2003).

Due to their ubiquitous presence on animal cells, integrins are commonly exploited by pathogens to mediate attachment and entry of virulence factors, bacterial cells, and viruses (Hussein et al., 2015; Stewart and Nemerow, 2007; Ulanova et al., 2009). The α_D_, α_L_, α_M_, and α_X_ integrin subunits pair exclusively with β_2_ and are expressed on leukocytes (Luo et al., 2007). The pore-forming RTX toxins (Linhartova et al., 2010), a family of related toxins secreted by the bacterial type I secretion system (T1SS), contains multiple members that use the β_2_-containing integrins as receptors (Ristow and Welch, 2019). Biochemical data suggest that these toxins interact with β_2_-containing integrins distal to the integrin ligand-binding site, with different toxins binding to different sites (Dileepan et al., 2007; Kieba et al., 2007; Lally et al., 1997; Li et al., 1999; Osicka et al., 2015; Ristow et al., 2019; Ristow and Welch, 2019; Shanthalingam and Srikumaran, 2009). Like all canonical T1SS substrates, pore-forming RTX toxins contain a C-terminal RTX domain comprising tandem repeats of a 9-residue calcium-binding motif that adopts a “β-roll” fold (Baumann et al., 1993; Welch, 1991) and mediates T1SS recruitment and secretion (Jarchau et al., 1994; Linhartova et al., 2010; Nicaud et al., 1986; Sebo and Ladant, 1993). Once secreted, the N-terminal portion of pore-forming RTX toxins inserts into target cell membranes and forms pores (Goebel and Hedgpeth, 1982; Linhartova et al., 2010). Pore-forming RTX toxins also contain a pair of conserved lysines that serve as acylation sites (Hackett et al., 1994; Stanley et al., 1994). Addition of an acyl group to at least one of these lysines, which is carried out by an acyltransferase enzyme encoded in the same operon as the toxin and secretion machinery, is essential for activity.

**Figure 1.**
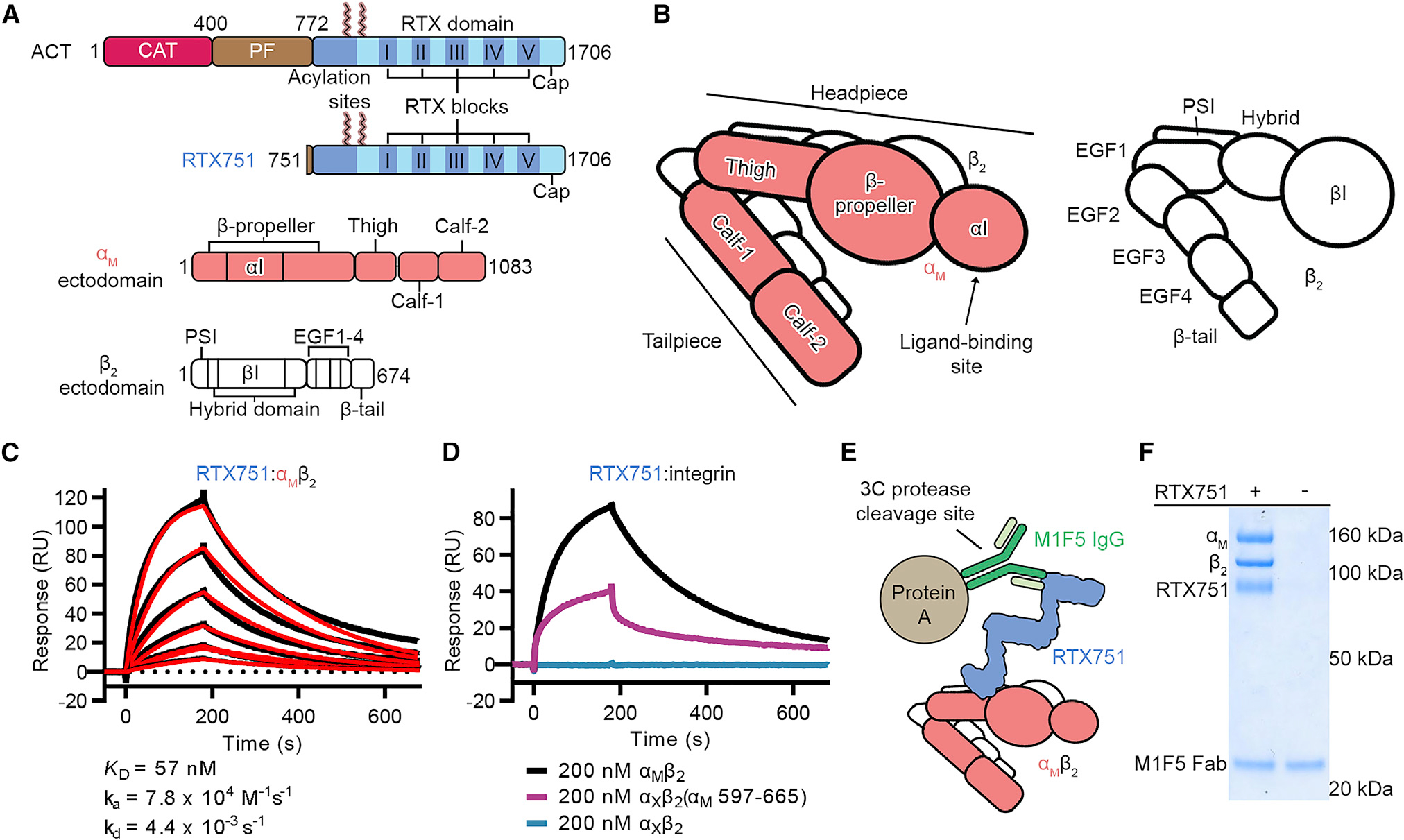
α_M_β_2_ interacts with RTX751 *in vitro* (A) Schematics of ACT and α_M_β_2_ protein domains. ACT is the full-length adenylate cyclase toxin secreted by *Bordetella*, and RTX751 is the recombinant C-terminal fragment used in this study. The adenylate cyclase domain is labeled CAT, and the pore-forming domain is labeled PF. Only the ectodomains are shown for α_M_ and β_2_ integrin subunits. (B) Diagram of the 3D organization of the α_M_ and β_2_ domains. α_M_β_2_ is depicted in the bent conformation. (C) Surface plasmon resonance measurement of RTX751:α_M_β_2_ binding kinetics. α_M_β_2_ association at varying concentrations was performed for 180 s, with a subsequent dissociation phase of 600 s. The binding data are shown in black, and red traces represent the best fit of the data to a 1:1 binding model. SPR experiments were performed a single time (N = 1) with one technical replicate of a single concentration. (D) Surface plasmon resonance measurements of immobilized RTX751 binding to different integrin constructs at 200 nM. Integrin association was performed for 180 s, with a subsequent dissociation phase of 600 s. Binding data for α_M_β_2_ are shown in black, α_X_β_2_ in teal, and α_X_β_2_(α_M_ 597–665) in purple. (E) Diagram showing the method used for co-immunoprecipitation of the α_M_β_2_-RTX751 complex. The 3C protease site is within the heavy-chain immunoglobulin G (IgG) hinge. (F) SDS-PAGE of the immunoprecipitation resulting from 3C protease-mediated elution.

Adenylate cyclase toxin (ACT) from *Bordetella pertussis*, the causative agent of whooping cough, is a member of the pore-forming RTX toxin family (Linhartova et al., 2010). However, unlike other pore-forming RTX toxins, ACT contains an N-terminal adenylate cyclase domain (Figure 1A). ACT inserts into target cells via its pore-forming domain (Figure 1A) and translocates the adenylate cyclase domain across the membrane by a poorly understood mechanism (Vojtova et al., 2006). Once in the host cytosol, activation of the adenylate cyclase by binding to calmodulin results in efficient catalysis that produces aberrantly high levels of cyclic AMP (Wolff et al., 1980). Through this intoxication mechanism, ACT disrupts a variety of leukocyte functions (Confer and Eaton, 1982; Gueirard et al., 1998; Kamanova et al., 2008; Khelef and Guiso, 1995; Pearson et al., 1987; Weingart and Weiss, 2000), promoting *Bordetella* colonization by preventing leukocyte-mediated clearance.

ACT uses the α_M_β_2_ integrin as a receptor, with binding required for efficient intoxication of macrophage J774A.1 cells as well as Chinese hamster ovary (CHO) cells recombinantly expressing integrin (Guermonprez et al., 2001; Osicka et al., 2015). The ACT binding site on α_M_β_2_ has been shown to include the thigh domain of α_M_ (Figure 1B), suggesting that ACT engages α_M_β_2_ using a non-canonical binding mode (Osicka et al., 2015). ACT was found to intoxicate cells more efficiently when α_M_β_2_ was in the inactive conformation (Osicka et al., 2015), but how ACT achieves this conformational specificity is not known. Although the α_M_β_2_-binding site has been localized to within residues 1,166–1,281 of the ACT RTX domain (El-Azami-El-Idrissi et al., 2003), no structural data for the binding of ACT to α_M_β_2_ are available, and its mode of non-canonical integrin engagement is poorly understood. In addition, the mechanism by which integrin binding facilitates membrane insertion and intoxication of cells is not known.

Here, we determine a 2.7Å resolution cryoelectron microscopy (cryo-EM) structure of the ACT receptor-binding domain in complex with the α_M_β_2_ ectodomain. This structure reveals the basis for non-canonical integrin engagement by ACT, as well as the preference of ACT for the inactive α_M_β_2_ conformation. In addition, the structure reveals a relationship between α_M_β_2_ binding and insertion of ACT into target cells, with binding positioning the conserved acylation sites at the host-cell membrane. The structure also provides a basis for the antibody-mediated inhibition of ACT, with implications for vaccine development.

## RESULTS

### α_M_β_2_ interacts with RTX751 *in vitro*

As it has been previously suggested that ACT interacts with α_M_β_2_ in the bent integrin conformation (Osicka et al., 2015), we purified integrin ectodomains containing an ACID/BASE C-terminal coiled-coil heterodimerization domain, which has been shown to favor the bent conformation in β_2_-containing integrins (Nishida et al., 2006; Xie et al., 2010). To assess whether the α_M_β_2_ ectodomain interacts with the purified RTX domain construct RTX751 (ACT residues 751–1,706), we performed surface plasmon resonance (SPR) measurements of α_M_β_2_ binding to immobilized RTX751. The α_M_β_2_ ectodomain bound to RTX751 with a K_D_ of 57 nM (Figure 1C), suggesting that the complex was sufficiently stable for cryo-EM studies. Consistent with cell-surface data, transfer of α_M_ thigh domain residues 597–665 onto α_X_β_2_, yielding, α_X_β_2_(α_M_ 597–665), rescued binding of the α_X_β_2_ ectodomain to RTX751 by SPR (Figure 1D). This result confirms that these residues of the α_M_ thigh domain are important for the interaction of the soluble protein components *in vitro*. However, α_X_β_2_(α_M_ 597–665) binding to RTX751 exhibited kinetics that deviated from a 1:1 model and exhibited a lower binding level than α_M_β_2_ (Figure 1D), which may be due to α_X_β_2_ (α_M_ 597–665) lacking additional α_M_ residues, partial misfolding of the chimera, or global conformational differences. Lastly, although RTX751 harbors the Lys860 and Lys983 acylation sites (Figure 1A), non-acylated RTX751 was used in this study. This suggests that the acyl modifications are not required for integrin binding.

### Cryo-EM structure of RTX751 in complex with α_M_β_2_ and M1F5 Fab

**Figure 2.**
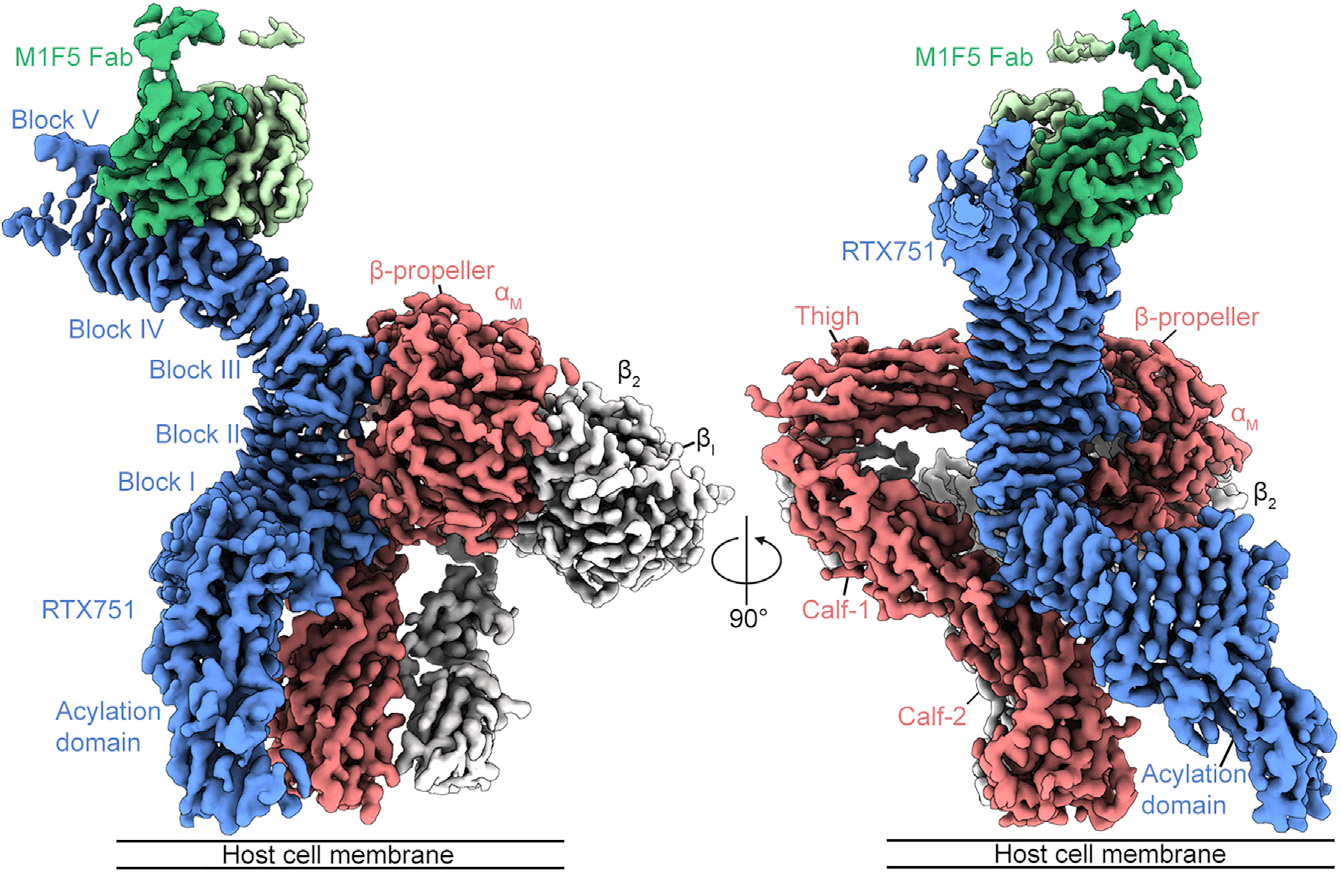
Cryo-EM structure of RTX751 in complex with α_M_β_2_ and M1F5 Fab Cryo-EM reconstruction with the α_M_ integrin subunit in pink, β_2_ integrin subunit in white, RTX751 in blue, M1F5 heavy chain in green, and M1F5 light chain in pale green. This composite map was generated in Phenix (Adams et al., 2002) by combining the reconstructions from the global refinement with those obtained using local refinement for the integrin tailpiece and RTX751 acylation domain. The component maps used to generate the composite were sharpened using DeepEMhancer (Sanchez-Garcia et al., 2021). See also Figures S1 and S2.

To purify the α_M_β_2_+RTX751 complex, we performed anti-RTX751 immunoprecipitation to ensure that all recovered α_M_β_2_ heterodimers were in a binding-competent state (Figure 1E). Immunoprecipitation was performed using the M1F5 antibody, as it does not inhibit ACT cell binding or intoxication and would not be expected to affect the RTX-α_M_β_2_ interaction (Wang et al., 2015). Binding of the M1F5 Fab to the complex also served to provide additional mass, which can improve cryo-EM particle alignment. SDS-PAGE confirmed that the immunoprecipitated ternary complex of α_M_β_2_+RTX751+M1F5 Fab exhibited the expected 1:1:1 stoichiometry (Figure 1F). The complex was then plunge-frozen on 1.2/1.3 UltrAuFoil grids and a dataset was collected on a Titan Krios using a K3 detector and a 30° stage tilt for 3,831 movies and 0° stage tilt for 500 movies. After motion correction and contrast transfer function estimation, two-dimensional (2D) classification revealed that the particle set contained a mix of the α_M_β_2_+RTX751+M1F5 Fab ternary complex and unbound α_M_β_2_. Subsequent 3D classification and heterogeneous refinement allowed us to separate the particles for the ternary complex from those of the unbound α_M_β_2_, yielding final particle sets of 347,508 particles for the ternary complex and 1,114,670 particles for unbound α_M_β_2_. Homogeneous refinement of these particle sets resulted in global 2.7 Å maps for each (Figure S1). However, in the global reconstructions for both the ternary complex and unbound α_M_β_2_, part of the integrin tailpiece was unresolved. Therefore, we used local refinement with a mask around the tailpiece to obtain maps that were more interpretable in this region (Figure S1). Similarly, the RTX acylation domain (residues 751–1,006) was poorly resolved, so local refinement with a mask around the acylation domain and RTX block I (residues 751–1,055) was performed (Figure S1). The set of global and local reconstructions for both the ternary complex (Figures 2 and S2) and unbound α_M_β_2_ (Figure S2) allowed for subsequent model building and refinement.

Docking of the crystal structure of ACT RTX blocks I–III (Goldsmith et al., 2021) into the ternary complex map allowed for unambiguous building of blocks I–IV of the RTX domain, although block V was unresolved (Figure 2). Notably, although the RTX domain in the published structure was in complex with receptor-blocking antibodies M2B10 and M1H5, it superimposes well with blocks I–III of α_M_β_2_-bound RTX751 (main-chain root-mean-square deviation [RMSD] 0.7 Å). This suggests that the RTX domain is rigid and that the α_M_β_2_-binding surface is unaffected by M1F5 binding. The majority of the ACT acylation domain was resolved, but the map contained breaks in the N-terminal ~70 residues that made model building difficult, and therefore a RoseTTaFold-predicted model was used to guide assignment of the register in this region (Baek et al., 2021). Overall, residues 754–1,488 of RTX751 were built, barring one unresolved loop (residues 1,355–1,369). To build the α_M_ integrin subunit, a crystal structure of α_X_β_2_ was used as a starting model (Xie et al., 2010). All of the residues of α_M_ were built, except for the αI domain (residues 123–331), which was unresolved due to flexibility relative to the β-propeller and two short unresolved loops in the calf-1 domain.

### ACT engages the β-propeller, thigh, and calf-2 domains of α_M_ via RTX linkers 1 and 2

**Figure 3.**
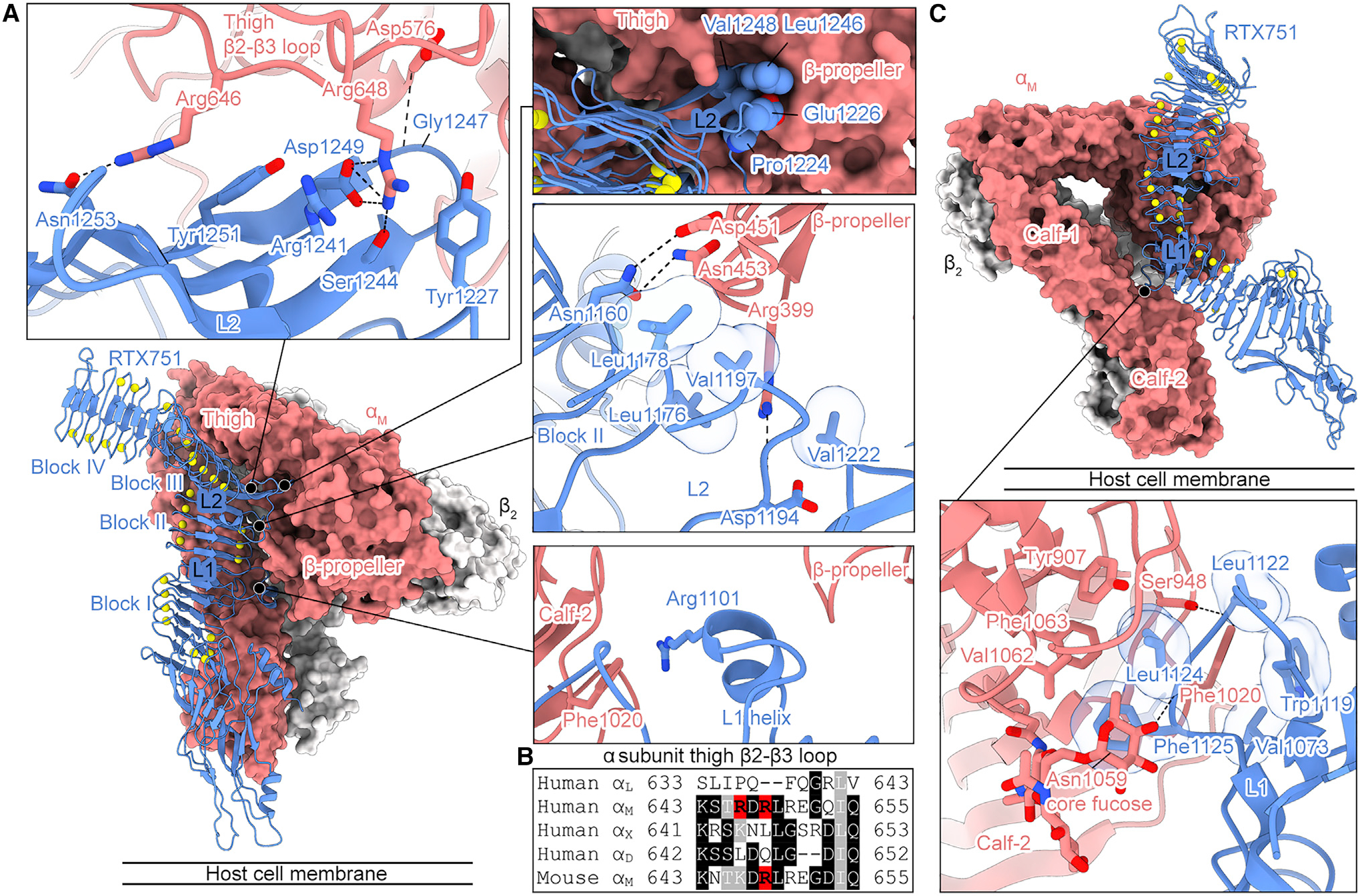
ACT engages α_M_ using RTX linkers 1 and 2 (A) Model of the RTX751-α_M_β_2_ complex. Insets show the interactions formed between α_M_ and ACT, with interface residues shown as sticks or spheres. Transparent molecular surfaces are shown for RTX751 side chains that form hydrophobic interactions. (B) Sequence alignment of the thigh β2-β3 loop from all human α integrin subunits that pair with β_2_, as well as mouse α_M_. Arg646 and Arg648 in human α_M_ are highlighted in red. (C) Interaction between the RTX751 linker 1 (L1) helix and the α_M_ calf-2 domain. Inset shows the interactions formed between α_M_ and ACT, with interface residues shown as sticks. Transparent molecular surfaces are shown for RTX751 side chains that form hydrophobic interactions. Oxygen atoms are colored red, nitrogen atoms are blue, and calcium ions are shown as yellow spheres. See also Figures S3 and S4.

The cryo-EM structure revealed that ACT forms an extensive interface with α_M_β_2_ that primarily involves the RTX domain linker regions between blocks I and II (L1) and between blocks II and III (L2) (Figures 3 and S3). This is consistent with L1 and L2 comprising the epitopes of the two known classes of neutralizing RTX-directed antibodies (Goldsmith et al., 2021; Wang et al., 2015). The α_M_β_2_-binding interface of RTX751 has a surface that is highly complementary to α_M_β_2_, resulting in a total buried surface area of 2,938 Å^2^. In contrast to canonical ligands, the RTX domain of ACT engages α_M_ without involving the ligand-binding αI domain. Instead, RTX751 inserts into the space between the bent α_M_ headpiece and tailpiece, and contacts the α_M_ β-propeller, thigh, and calf-2 domains (Figure 3A). ACT can only contact both the α_M_ headpiece (containing the β-propeller and thigh domains) and the α_M_ calf-2 simultaneously if the integrin is bent. This finding provides the structural basis for the specificity of ACT for the bent α_M_β_2_ conformation, which was previously hypothesized (Osicka et al., 2015).

The α_M_ thigh domain is required for binding by RTX751 (Osicka et al., 2015), suggesting it contains a critical portion of the binding interface. Despite most of the thigh domain being too far from RTX751 for contact, the thigh β2-β3 loop extends toward the α_M_ β-propeller and interacts with RTX751 L2 (Figure 3A). Arg648 of the thigh β2-β3 loop forms a salt bridge with RTX751 Asp1249, a hydrogen bond with RTX751 Ser1244, and π-π stacking interactions on both faces with RTX751 Tyr1227 and Arg1241 (Figure 3A). In addition, Arg646 of the α_M_ thigh β2-β3 loop forms a hydrogen bond with RTX751 Asn1253 and a hydrophobic interaction between its aliphatic region and the RTX751 Tyr1251 side chain (Figure 3A). The extensive contacts formed by these two key arginine residues with RTX751 L2 explain why the thigh β2-β3 loop is essential for α_M_β_2_ binding (Osicka et al., 2015). In addition, the β2-β3 loops of α_X_, α_L_, and α_D_ do not contain these arginine residues (Figure 3B), providing a basis for the specificity of ACT for α_M_β_2_ and not the other β_2_-containing integrins (Guermonprez et al., 2001; Osicka et al., 2015).

Aside from the interaction with the α_M_ thigh β2-β3 loop, RTX751 contacts regions of α_M_β_2_ that are largely conserved between α_M_ and α_X_ (Figure S4A), which likely explains why ACT binding to α_X_β_2_ can be rescued upon inclusion of only the α_M_ thigh domain (Osicka et al., 2015). Within the integrin headpiece, RTX751 contacts the α_M_ β-propeller domain with L2 as well as with part of RTX block II (Figure 3A). The helix-containing loop in RTX751 L1 also inserts into the space between the α_M_ β-propeller and calf-2 (Figure 3A). RTX751 forms an interface with the α_M_ calf-2 domain primarily involving L1 that is largely hydrophobic (Figure 3C). One notable difference between α_M_ and α_X_ is that α_M_ Phe1020, which forms hydrophobic and π-π stacking interactions with RTX751, is a serine in α_X_ (Ser1017) (Figure S4A). Therefore, the interactions formed by Phe2010 are not critical for ACT binding but may still contribute to the improved binding of α_M_β_2_ relative α_X_β_2_(α_M_ 597–665) (Figure 1D). The ternary complex map also contained a well-resolved N-linked glycan at Asn1059 in α_M_ calf-2 (Figure 3C), with the core fucose residue of the glycan packing onto RTX751 Leu1124 and Phe1125. Notably, glycans have been shown to be important for ACT binding to α_M_β_2_-expressing cells (Hasan et al., 2015; Morova et al., 2008). Furthermore, investigation of the effects of site-specific glycan mutants showed that ablation of the Asn1059 glycan resulted in a 2-fold decrease in ACT binding, the largest effect among all of the glycans tested (Hasan et al., 2015). However, α_M_ Asn1059 is substituted for Asp1056 in α_X_ (Figure S4A), suggesting that this glycan is not strictly required for ACT binding. Overall, these results show that ACT engages α_M_β_2_ using a binding mode that differs from typical integrin ligands, whereby an extensive interface is formed with three different domains on the side of the bent α_M_ subunit.

*B. pertussis* ACT can efficiently intoxicate J774A.1 murine macrophage cells in a manner that depends on α_M_β_2_ binding (Guermonprez et al., 2001; Osicka et al., 2015; Wang et al., 2015), and ACT is essential for lung colonization in infant mouse models of *B. pertussis* infection (Goodwin and Weiss, 1990; Weiss et al., 1984). These data suggest that mouse α_M_β_2_ is sufficiently similar to human α_M_β_2_ to allow for ACT binding. Analysis of the ACT-binding interface showed that the integrin residues contacting ACT are almost entirely conserved in mouse α_M_β_2_. One notable difference is that Arg646 is substituted for Lys646 in the mouse α_M_ thigh β2-β3 loop (Figures 3B and S4B), suggesting that lysine at this position can form similar interactions with RTX751 L2. In addition, just as in α_X_, α_M_ Phe1020 is substituted for a serine in the calf-2 domain of mouse α_M_ (Ser1021) (Figure S4B). This further corroborates that the hydrophobic and π-π stacking interactions formed by Phe1020 are not strictly required for α_M_β_2_ binding by ACT. The only other non-conserved ACT-binding residue between human and mouse α_M_ is the substitution of Asn453 for Asp451 in mouse α_M_ (Figure S4B). However, the aspartate at this position should still form a hydrogen bond with Asn1160 in RTX block II. Thus, the highly conserved ACT-binding interface between human and mouse α_M_ explains the cross-species reactivity of ACT.

### ACT traps a partially extended α_M_β_2_ conformation

Previous studies have shown that integrins are flexible in the bent conformation, with multiple copies of α_X_β_2_ in the asymmetric unit of a crystal structure exhibiting varying inter-domain angles (Xie et al., 2010). Consistent with these observations, the integrin tailpiece in the cryo-EM reconstruction of unbound α_M_β_2_ was poorly resolved (Figure S2B). In contrast, the α_M_β_2_ tailpiece was substantially better resolved in the reconstruction of the ternary complex (Figure S2A), likely due to RTX751 stabilizing multiple domains of α_M_.

**Figure 4.**
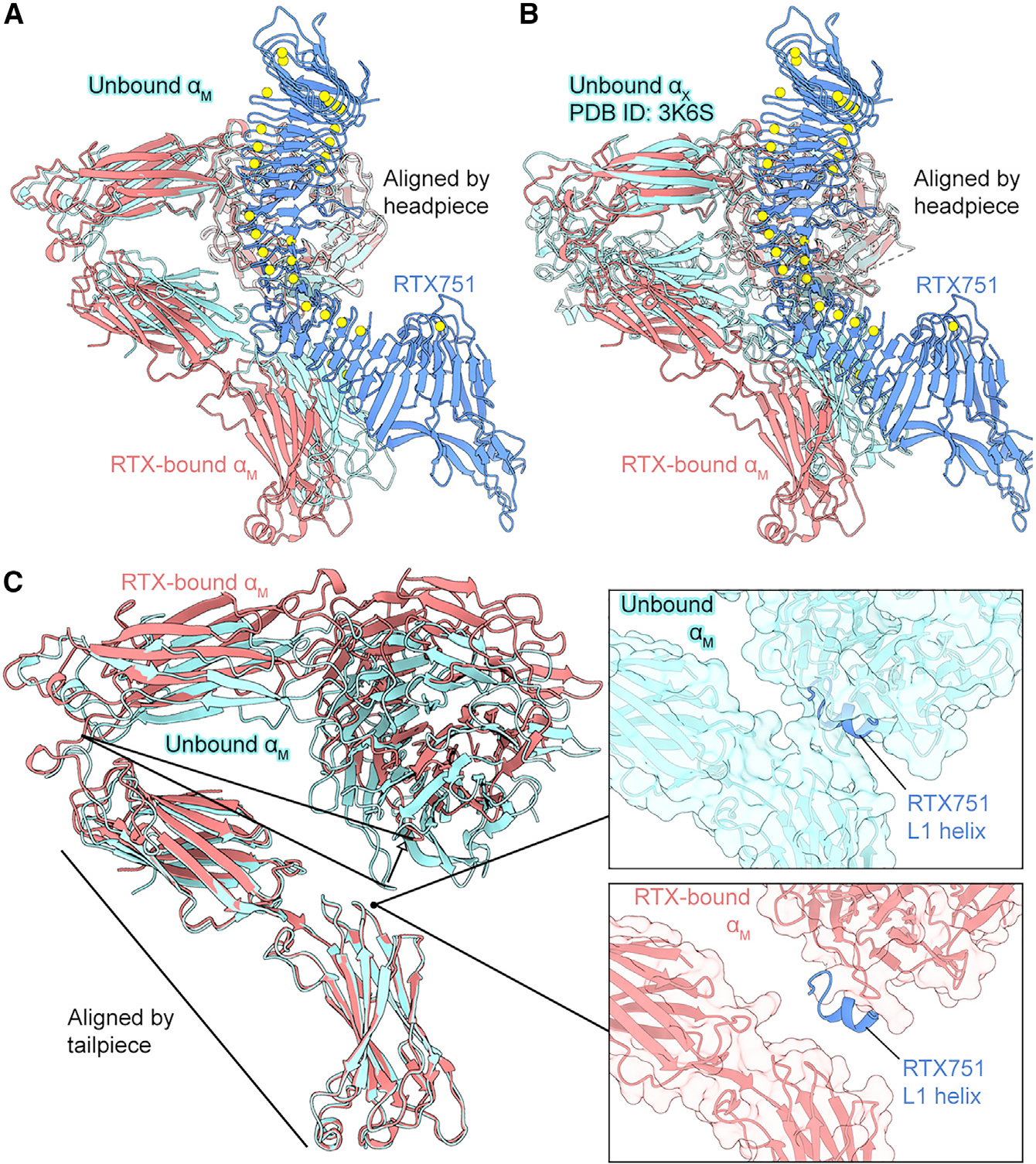
ACT traps a partially extended α_M_β_2_ conformation (A and B) Structure of α_M_ bound to RTX751 superimposed with either (A) the unbound α_M_ model derived from the same cryo-EM dataset, or (B) α_X_ from the crystal structure of α_X_β_2_ (PDB: 3K6S) based on alignment of the headpieces. RTX751 is shown in blue, with calcium ions shown as yellow spheres. RTX-bound α_M_ is colored pink, while unbound α_M_ or α_X_ are shown in cyan. (C) RTX-bound α_M_ and unbound α_M_ aligned by the tailpiece to allow visualization of the hinge motion of the headpiece. α_M_ models are shown as ribbons, along with a transparent molecular surface in the insets. Insets show the position of the RTX751 L1 helix from the RTX751-α_M_β_2_ complex as a ribbon.

Comparison of unbound α_M_β_2_ with RTX751-bound α_M_β_2_ showed that the binding of the RTX domain stabilizes a conformation of α_M_β_2_ that is partially extended relative to the unbound α_M_β_2_ (Figure 4A). RTX751-bound α_M_β_2_ is similarly extended relative to the available α_X_β_2_ crystal structures (Sen et al., 2013; Xie et al., 2010) (Figure 4B). The bound and unbound α_M_β_2_ are related by a hinge motion of the headpiece relative to the tailpiece (Figure 4C). As L2 interacts with the thigh/β-propeller in the α_M_ headpiece and L1 interacts with the calf-2 domain in the tailpiece, the rigid RTX domain connecting L1 and L2 stabilizes a fixed distance between the thigh/β-propeller and calf-2 of α_M_. The position of the RTX751 L1 helix between the α_M_β_2_ headpiece and tailpiece would result in a steric clash with α_M_ in the unbound conformation (Figure 4C), meaning the L1 helix may also contribute to the stabilization of the partially extended state. Although the unbound α_M_β_2_ adopts a fully bent conformation similar to that observed in bent α_X_β_2_ crystal structures (Sen et al., 2013; Xie et al., 2010), the ability of RTX751 to bind α_M_β_2_ suggests that it transiently adopts greater headpiece-tailpiece hinge angles in solution, consistent with previous observations (Beglova et al., 2002; Takagi et al., 2002).

### α_M_β_2_ binding positions the ACT acylation sites at the cell membrane

**Figure 5.**
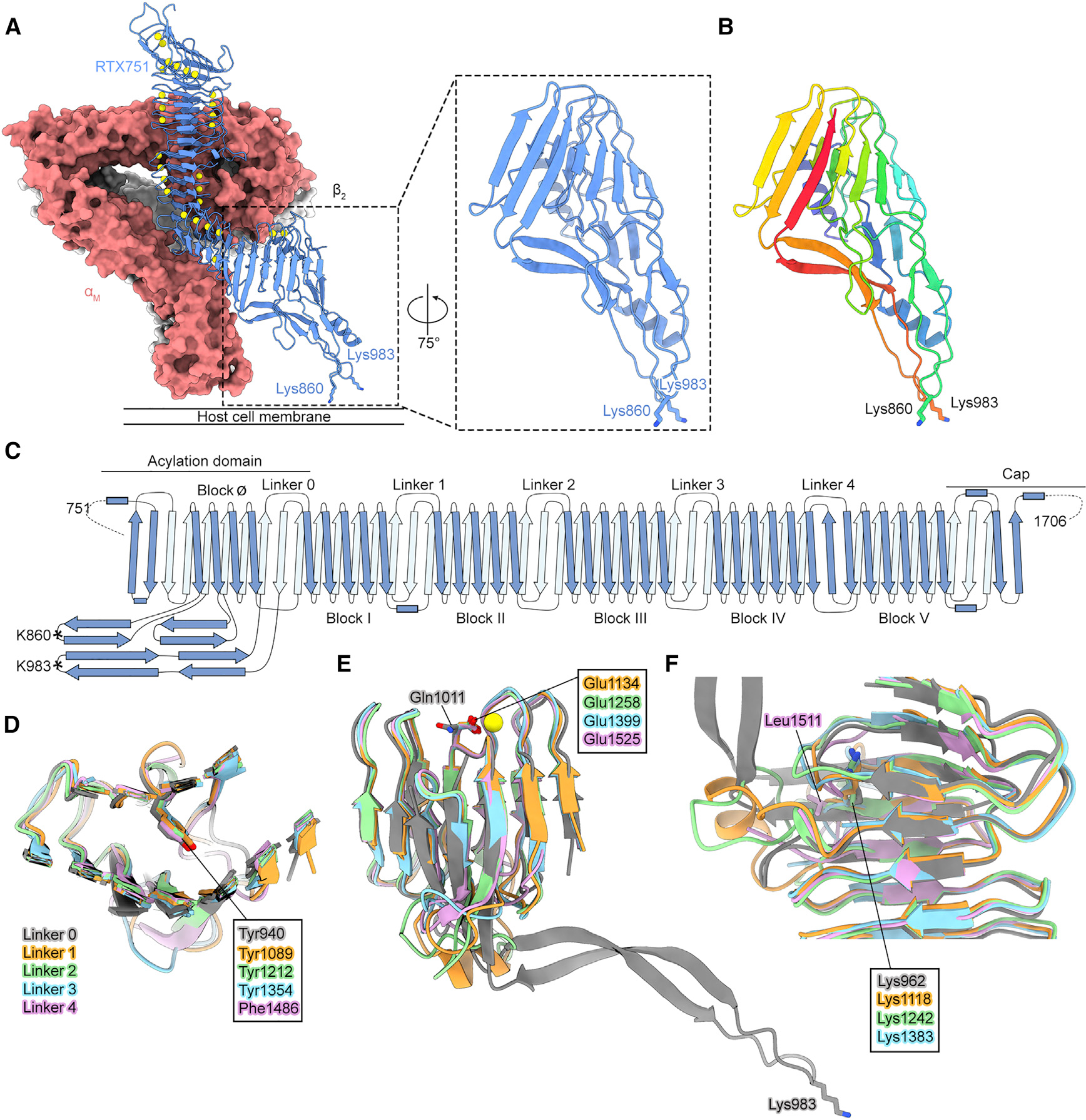
The ACT acylation sites are positioned at the cell membrane (A) RTX751 in complex with α_M_β_2_. Calcium ions are shown as yellow spheres, and the side chains of Lys860 and Lys983 acylation sites are shown as sticks with nitrogen atoms colored dark blue. The approximate location of the host cell membrane is labeled. A zoomed-in view of the acylation domain is shown in the dashed box. (B) ACT acylation domain with rainbow coloring from blue to red, N to C terminus. (C) Topology diagram depicting the organization of the ACT RTX domain. β strands are shown as arrows, and α helices are shown as rectangles. β strands on the opposite face of the β-roll are colored pale blue. (D–F) Structural alignment of the inter-block linkers of the RTX domain. Residues at conserved positions within the linker motif are shown as sticks, with oxygens colored red, nitrogens colored blue, and calcium ions shown as yellow spheres. Residues that conform to the RTX linker consensus motif are labeled in boxes, whereas residues deviating from the consensus are labeled directly on the residue. (D) Conserved tyrosine/phenylalanine. (E) Conserved glutamate. (F) Conserved internal lysine. See also Figure S5

The structure of RTX751 shows that the acylation domain of RTX toxins is a continuation of the β-roll fold of the RTX domain, with the two acylation sites (Lys860 and Lys983) located at the tips of long loops that protrude from the β-roll core (Figure 5A). Although the acylation sites in ACT are separated by 123 residues, they are positioned at the tips of adjacent loops (Figure 5B). This proximity likely explains the partial redundancy that has been observed between these two sites for ACT (Masin et al., 2005) and suggests that their precise positioning is important for RTX toxin activation. Notably, ACT binding to α_M_β_2_ positions Lys860 and Lys983 at the plane of the host-cell membrane, with both side chains pointing toward the membrane (Figure 5A). This suggests that the essential acylations in pore-forming RTX toxins are involved in direct insertion into the target membrane.

The structure also revealed an evolutionary relationship between the conserved acylation domain of pore-forming RTX toxins and linkers 1–4 of *B. pertussis* ACT. We previously observed that the linker regions between RTX blocks of ACT adopt a conserved fold and contain specific conserved residues, suggesting that they arose through the duplication of a single linker (Goldsmith et al., 2021). The β-roll structure of the acylation domain contains the same higher-order organization as RTX blocks I–V: An RTX block (called block Ø) followed by a linker with the conserved fold observed in L1–L4 (Figure 5C). The linker within the acylation domain, called linker 0 or L0, contains Tyr940 at the position of a strictly conserved core-facing tyrosine or phenylalanine (Figure 5D). While L0 contains a glutamine at the position of the conserved glutamate in L1–L4 (Figure 5E), alignment of the *B. pertussis* L0 to L0 from a set of well-studied members of the pore-forming RTX toxin family showed that all other pore-forming RTX toxins analyzed contain the conserved glutamate (Figure S5). Notably, L0 contains a buried lysine side chain (Lys962) that is also present in L1, L2, and L3 (Figure 5F). In all four instances of this buried lysine, the ε-amino group occupies the position within the β-roll that typically would contain a calcium ion (Figure 5E). Surprisingly, this lysine is substituted for arginine in the acylation domains of other pore-forming RTX toxins (Figure S5). As the residues surrounding the lysine ε-amino group would clash with the guanidinium group of an arginine at this position, the acylation domain of pore-forming RTX toxins with this arginine likely form a bulge to accommodate it. Although the core β sheets of L0–L4 adopt the same fold, the key difference between L0 in the acylation domain and L1–L4 is that L1–L4 contain shorter variable loops and L0 contains a much longer loop harboring the Lys983 acylation site (Figures 5C and S5). The conservation between L0 and L1–L4 suggests that they share a common ancestor. Because L0 is conserved among the pore-forming RTX toxins that generally do not contain the additional linkers seen in ACT, we conclude that L0 is the ancestral linker, and that L1–L4 of ACT arose through the duplication of L0.

### Inter-block linkers are immunogenic sites within the ACT RTX domain

M1F5 binds L3 between blocks III and IV (Figure S6), similar to how M2B10 binds L1 and M1H5 binds L2 (Goldsmith et al., 2021). M1F5 forms 2 π-π stacking interactions with L3, 5 hydrogen bonds with L3, 2 π-π stacking interactions with block IV, and 4 hydrogen bonds with block IV (Figure S6A). Thus, all 3 RTX domain antibodies whose epitopes have been mapped by high-resolution structures interact with the RTX linkers (Figure S6B). This suggests that the linkers are particularly immunogenic relative to the RTX blocks.

The ternary complex structure also revealed the basis for ACT neutralization by anti-RTX antibodies M2B10 and M1H5. Consistent with their prevention of α_M_β_2_ binding, both M2B10 binding to L1 and M1H5 binding to L2 would result in a steric clash with α_M_β_2_ (Figure S6B). By contrast, M1F5 is non-neutralizing as its epitope is not part of the α_M_β_2_-binding interface (Figure S6B). Interestingly, M1H5 mimics Arg648, the key interface residue in the α_M_ thigh β2-β3 loop (Figure S6C). The guanidinium group of M1H5 CDRH3 Arg98 is in the same position as α_M_ Arg648, such that they each form a salt bridge with Asp1249, hydrogen bond with Ser1244, and π-π stack with Tyr1227 and Arg1241 in RTX751 L2 (Figure S6C). This suggests that M1H5-like antibodies against ACT may be resistant to escape.

## DISCUSSION

Canonical integrin ligands form high-affinity interactions with the MIDAS that involve a specific consensus “RG” tripeptide in the ligand. Many integrin-mediated host-pathogen interactions involve the binding of a pathogen-encoded RGD motif to the integrin MIDAS. In addition, the entry or attachment mechanism often specifically necessitates integrin activation that results from RGD binding to the MIDAS. For example, adenoviruses such as Ad2 and Ad12 use α_V_β_5_ and α_V_β_3_ as entry receptors (Wickham et al., 1993, 1994). In these viruses, an RGD motif that protrudes from a loop in the penton base of the viral capsid binds to the integrin MIDAS and drives activation and intracellular signaling, with the downstream stimulation of endocytosis facilitating viral entry (Chiu et al., 1999). By contrast, echovirus 1 binds the α_2_β_1_ αI domain, but not at the ligand-binding site and in an RGD-independent manner (Jokinen et al., 2010). This RGD-independent binding mode has no preference for the active or inactive conformation and does not drive intracellular signaling. In bacteria, *Yersinia* invasin directly binds to β_1_-containing integrins using an RGD-like motif to mediate cell internalization (Isberg and Leong, 1990; Leong et al., 1995), with *Neisseria meningitidis* NadA likely functioning by a similar mechanism (Nagele et al., 2011). Secreted virulence factors also use integrin receptors, with an RGD motif in the *Helicobacter pylori* T4SS component CagL binding to α_5_β_1_, allowing for transport of the T4SS cargo into the host cell (Kwok et al., 2007). Similarly, the staphylococcal toxin LukGH uses an RGD to bind to α_M_β_2_, with activation-induced integrin clustering being required for LukGH function (DuMont et al., 2013; Trstenjak et al., 2020).

The structure of RTX751 in complex with α_M_β_2_ reveals a structural mechanism by which the inactive integrin conformation can be targeted. Namely, the headpiece and tailpiece of α_M_β_2_ must adopt the hinge angle corresponding to the bent integrin conformation to form the full ACT-binding site. Given the importance of the α_M_ thigh β2-β3 loop for ACT binding, it is conceivable that RTX L2 could bind to the headpiece of extended α_M_β_2_ on the host cell surface without forming the interface between L1 and the α_M_ calf-2. However, receptor-blocking antibodies M2B10 and M1H5 similarly neutralize ACT intoxication of J774A.1 murine macrophages (Wang et al., 2015), even though M2B10 prevents the binding of α_M_ calf-2 by RTX L1 and is not expected to clash with α_M_ in the extended conformation. This suggests that α_M_β_2_ binding to calf-2 in the bent conformation is required at some point during intoxication.

Hantaviruses have also been shown to preferentially engage the bent α_V_β_3_ conformation (Raymond et al., 2005), but the nature of this conformational specificity is unclear. This is because the hantaviral binding site is the PSI domain in the β_2_ legs, whose precise arrangement in the extended conformation is not known. The α_M_β_2_-binding mode of ACT suggests that a plausible mechanism of bent specificity for hantaviruses is that it could form additional contacts with the lower integrin legs, or other domains that are only sufficiently close in the bent conformation. Interestingly, a designed disulfide bond that favors hantaviral entry (Raymond et al., 2005) is expected to trap the fully bent α_V_β_3_ conformation, with the smallest possible headpiece-tailpiece angle being required for its formation. Such a disulfide bond in α_M_β_2_ would likely preclude ACT binding, which requires partial extension relative to the fully bent conformation.

The observation that ACT uses L1 and L2 to engage α_M_β_2_ shows that integrin binding evolved independently in ACT, as typical pore-forming RTX toxins that are more similar to *Escherichia coli* HlyA do not contain the RTX linker motif outside of L0 in the acylation domain. HlyA, which uses α_L_β_2_ as a receptor (Lally et al., 1997), has a much shorter RTX domain consisting of only the acylation domain (block Ø and linker 0), one RTX block, and the C-terminal cap conserved among T1SS substrates (Bumba et al., 2016; Linhartova et al., 2010). However, adapting the inter-block linkers for protein-protein interactions is intuitive as the flat β-roll formed by the calcium-binding repeats is expected to have limited capacity to form a complementary binding surface. Therefore, the absence of inter-block linkers in other pore-forming RTX toxins points to a mystery as to how they bind their integrin receptors. It is possible that some other pore-forming RTX toxins engage β_2_-containing integrins using the N-terminal pore-forming domain. However, swapping the ACT RTX domain for the RTX domain of *E. coli* HlyA altered the specificity of ACT such that it required α_L_β_2_ instead of α_M_β_2_ on the target cell (Masin et al., 2020). Thus, at least some pore-forming RTX toxins may bind to integrin receptors via RTX domains that lack linker modules. In addition, the conservation of the Lys860 and Lys983 acylation sites and the requirement that they are acylated for toxin function suggest that their mechanistic role is conserved across members of the family. Therefore, it is likely that the convergent mechanisms by which pore-forming RTX toxins bind β_2_-containing integrins also result in their positioning at the membrane. Further structural studies will be required to understand how integrin binding is achieved by other pore-forming RTX toxins.

As the acylation domain contains the conserved linker motif previously observed between RTX blocks I–V (Goldsmith et al., 2021; Motlova et al., 2020), ACT linkers 1–4 likely arose via initial duplication of linker 0. In general, formation of a receptor-binding surface likely provided the selective advantage driving expansion of the ACT RTX domain. However, the ACT receptor-binding site comprises only L1 and L2, and recent work showed that ACT can intoxicate cells as efficiently as wild type when the region of the RTX domain containing L3 and L4 is deleted (Espinosa-Vinals et al., 2021). Therefore, it remains a question as to whether L3 and L4 have any function, potentially involving protein-protein interactions. As the linkers appear to be particularly immunogenic, it is possible that L3 and L4 could bias anti-ACT antibody responses toward non-neutralizing epitopes. In addition, the linker motif in the acylation domain provides a rationale for the observation of impaired membrane insertion and intoxication by ACT containing the mutation Y940A (Masin et al., 2017). This residue is located at the position of the strictly conserved core-facing tyrosine/phenylalanine in the N-terminal portion of the linker motif (Goldsmith et al., 2021). Removal of the phenyl ring by substitution to alanine would therefore be expected to disrupt folding of the linker motif, which could alter the positioning of the Lys983 acylation and preclude efficient membrane insertion. By contrast, the observation that Y940F does not disrupt ACT membrane insertion or intoxication is consistent with the tolerance of the linker motif to either tyrosine or phenylalanine at this position, as exemplified by Phe1486 in L4. A recent study also assessed the effect of alanine substitutions at this position in other pore-forming RTX toxins. Given the importance of this residue in ACT, it is striking that its mutation did not significantly affect the hemolytic activities of *E. coli* HlyA, and only modestly affected *Kingella kingae* RtxA, or *Actinobacillus pleuropneumonia* ApxIA (Lepesheva et al., 2021). This suggests that the pore-forming RTX toxins more similar to *E. coli* HlyA may have more stable acylation domains than ACT that can maintain the structure of the loop harboring the acylation site homologous to ACT Lys983 despite local unfolding of L0.

In conclusion, we found that ACT achieves specificity for the bent α_M_β_2_ conformation by interacting with both the headpiece and tailpiece, which separate during extension. This binding mode differs from other known conformation-specific ligands and monoclonal antibodies and represents a strategy by which pathogens can attach to integrins without favoring activation. Binding to the bent α_M_β_2_ conformation also positions the essential acyl groups of ACT at the cell membrane, coupling receptor binding to membrane insertion. These findings will aid in the design of ACT-based reagents that report or induce the bent α_M_β_2_ conformation, as well as ACT-based immunogens for next-generation pertussis vaccines.

### Limitations of the study

The main limitation of this study is that it only used an *in vitro* reconstituted system to study the binding of the soluble α_M_β_2_ ectodomain to RTX751, whereas during the infection of animals, ACT binds membrane-embedded α_M_β_2_ on the surface of leukocytes. Therefore, the affinity reported for the interaction of the soluble proteins (57 nM) may not reflect the affinity *in vivo*, where ACT must find α_M_β_2_ by sampling a 2D surface. Without the cell or membrane environment, additional structural features of the binding and insertion mechanism may be missed. In addition, the propensity of our α_M_β_2_ construct to adopt the bent conformation may affect RTX751 binding, potentially either by improving association due to preventing extension, or inhibiting association by favoring the binding-incompatible fully bent state. The conclusions of this study could also be bolstered further by a systematic mutagenesis-based dissection of individual residue contributions, as well as the optimization of techniques to precisely modulate the integrin conformational state.

## STAR★METHODS

### RESOURCE AVAILABILITY

#### Lead contact

Further information and requests for resources and reagents should be directed to and will be fulfilled by the Lead Contact, Jason McLellan jmclellan@austin.utexas.edu.

#### Materials availability

All unique/stable reagents generated in this study are available from the Lead Contact with a completed Materials Transfer Agreement.

#### Data and code availability

Atomic coordinates for α_M_β_2_ in complex with RTX751 and M1F5 Fab and for unbound α_M_β_2_ have been deposited into the Protein Data Bank and assigned PDB IDs 7USL and 7USM, respectively. Cryo-EM maps for α_M_β_2_ in complex with RTX751 and M1F5 Fab have been deposited in the EMDB and assigned codes EMD-26738 (composite map), EMD-27122 (global refinement map), EMD-27123 (tailpiece local refinement map), and EMD-27124 (acylation domain local refinement map). Cryo-EM maps of unbound α_M_β_2_ have been deposited in the EMDB and assigned codes EMD-26739 (composite map), EMD-27125 (global refinement map), and EMD-27126 (tailpiece local refinement map).

This paper does not report original code.

Any additional information required to reanalyze the data reported in this paper is available from the Lead Contact upon request.

### EXPERIMENTAL MODEL AND SUBJECT DETAILS

FreeStyle 293-F mammalian cells (ThermoFisher) were maintained in FreeStyle 293 expression medium (Gibco) in a 37°C shaker supplied with 8% CO_2_ and 80% humidity. These cells were purchased directly from ThermoFisher, and no further authentication was performed.

### METHOD DETAILS

#### Protein expression and purification

A gene encoding RTX751 (ACT residues 751–1706) was cloned into pET22b downstream of an N-terminal 8xHis tag and HRV 3C protease recognition site. This construct was then transformed into *E. coli* BL21 DE3 for protein expression. Cells at OD600 = 0.6 were induced with 1 mM IPTG and grown for 16h at 16°C. Following growth, cells were centrifuged for 20 min at 5,000×g. Cells were then resuspended in 50 mM Tris pH 8, 200 mM NaCl, 2 mM CaCl2, 10 mM imidazole and passed twice through a Microfluidics LM10 microfluidizer at 18,000 psi. Lysate was then centrifuged for 1h at 30,000×g and 4°C. NiNTA resin equilibrated with 50 mM Tris pH 8, 200 mM NaCl, 2 mM CaCl2, 10 mM imidazole was then magnetically stirred with the soluble fraction for 30 min at 4°C. The resin was then washed on-column with 50 mM Tris pH 8, 200 mM NaCl, 2 mM CaCl2, 40 mM imidazole. Elution was performed with 50 mM Tris pH 8, 200 mM NaCl, 2 mM CaCl2, 150 mM imidazole. The elution was then concentrated, run over a Superdex 200 10/300 gel-filtration chromatography column (Cytiva), concentrated, and flash-frozen using liquid N2 until use.

Genes encoding α_M_ residues 1–1104 (including native signal sequence), α_X_ residues 1–1103 (including native signal sequence), or α_X_ residues 1–1103 (including native signal sequence) with residues 614–682 (595–663 in mature ectodomain) replaced with residues 613–681 (597–665 in mature ectodomain) of α_M_, were cloned into pαH upstream of a disulfide bond-forming linker (GCGG) (Xie et al., 2010), an HRV 3C cleavage site (LEVLFQGP), an ACID coiled-coil heterodimerization motif (GENAQCEKELQALEKENAQLEWELQALEKELAQ) (Nishida et al., 2006) and two StrepTagII tags separated by a GlySer linker. A gene encoding β2 residues 1–699 (including native signal sequence) was cloned into pαH upstream of a disulfide bond-forming linker (DGCG) (Xie et al., 2010) followed by an HRV 3C cleavage site (LEVLFQGP), a BASE coiled-coil heterodimerization motif (GKNAQCKKKLQALKKKNAQLKWKLQALKKKLAQGG) (Nishida et al., 2006) and a 6xHis tag. Plasmid encoding α_M_, α_X_ or α_X_(α_M_ 597–665) ectodomain was transiently co-transfected with plasmid encoding β2 ectodomain into FreeStyle 293-F cells (Invitrogen) using polyethylenimine (Polysciences). After 6 days, the cell supernatant was harvested and passed through a 0.22 μM filter. Filtered supernatant was then buffer-exchanged into 20 mM Tris pH 7.5, 150 mM NaCl, 1 mM CaCl_2_, 1 mM MgCl_2_ using tangential flow filtration. Integrin heterodimer was then purified from buffer-exchanged supernatant using StrepTactin XT resin equilibrated with 20 mM Tris pH 7.5, 150 mM NaCl, 1 mM CaCl_2_, 1 mM MgCl_2_. Resin was washed with 20 mM Tris pH 7.5, 150 mM NaCl, 1 mM CaCl_2_, 1 mM MgCl_2_ and protein was eluted with 20 mM Tris pH 7.5, 150 mM NaCl, 1 mM CaCl_2_, 1 mM MgCl_2_, 50 mM biotin. The elution was then concentrated, run over a Superdex 200 10/300 gel-filtration chromatography column (Cytiva), concentrated, and flash-frozen using liquid N2 until use.

To express M1F5 IgG, M1H5 IgG, or anti-StrepTagII IgG, plasmid encoding the light chain was transiently co-transfected with plasmid encoding the heavy chain (containing an HRV 3C protease cleavage site in the hinge region) into FreeStyle 293-F cells (Invitrogen) using polyethylenimine (Polysciences). After 6 days, the supernatant was harvested and passed through a 0.22 μm filter. IgG was then purified from filtered supernatant using Protein A Agarose (ThermoFisher). Protein A elutions containing IgG were then dialyzed against 20 mM Tris pH 7.5, 150 mM NaCl, 2 mM CaCl_2_ before being flash-frozen using liquid N_2_ and stored until use. To generate M1H5 Fab for biolayer interferometry, M1H5 IgG was incubated with 1:20 wt/wt HRV 3C protease overnight at 4°C. Fc was then removed by passing the cleavage reaction over Protein A Agarose (ThermoFisher), after which the Fab was further purified using a Superdex 200 10/300 gel-filtration chromatography column (Cytiva). Fab was then flash-frozen using liquid N2 and stored until use.

#### Surface plasmon resonance

For coupling, M1F5 Fab was desalted using a HiPrep 26/10 desalting column (Cytiva) into 10 mM Na-acetate pH 4.0 and concentrated to 1 mg/mL. Using an EDC/NHS amine coupling kit (Cytiva), ~2500 RU M1F5 Fab was coupled onto both flow cells of a CM5 SPR biosensor (Cytiva). Kinetic measurements were performed in a running buffer of 10 mM HEPES pH 7.4, 150 mM NaCl, 1 mM CaCl_2_ 1 mM MgCl_2_, and 0.05% Tween-20. For each cycle of the α_M_β_2_ concentration series, ~70 RU RTX751 (10 nM, 5s flow rate, 15s) was coupled onto flow cell 2. Then, buffer (for double-reference subtraction) or α_M_β_2_ at 6.13 nM, 12.5 nM, 25 nM, 50 nM, 100 nM, or 200 nM was flowed into both flow cells to measure association for 180s. Dissociation was measured for 600s while flowing in running buffer and data were fit to a global 1:1 binding model for determination of kinetic parameters. For the single-concentration comparison of integrin heterodimer binding ~60 RU RTX751 (10 nM, 5s flow rate, 15s) was coupled onto flow cell 2. Then, buffer (for double-reference subtraction), 200 nM αMβ2, 200 nM αXβ2 or 200 nM α_X_β_2_(αM 597–665), was flowed into both flow cells to measure association for 180s. Running buffer was subsequently flowed in for 600s to measure dissociation.

#### α_M_β_2_+RTX751+M1F5 immunoprecipitation

To form a complex of αMβ2+RTX751+M1F5 IgG, 150 μg αMβ2, 25 μg RTX751, and 20.6 μg M1F5 IgG (1:1:1 molar ratio of αMβ2:RTX751:M1F5 Fab) were mixed in a total volume of 140 μL and allowed to bind at room temperature for 20 min 120 μL of Protein A Agarose (Thermo Fisher) was then equilibrated 3 times in a microcentrifuge tube by mixing with 1 mL 20 mM Tris pH 7.5, 150 mM NaCl, 1 mM CaCl_2_, 1 mM MgCl_2_, centrifuging, and removing the supernatant. The binding reaction was then mixed with the resin, which was subsequently centrifuged, and the supernatant was removed. To wash the resin, it was mixed with 1.4 mL of 20 mM Tris pH 7.5, 150 mM NaCl, 1 mM CaCl_2_, 1 mM MgCl_2_, which was then centrifuged and decanted. To elute the ternary complex, 1 μg of HRV 3C protease was added to 120 μL 20 mM Tris pH 7.5, 150 mM NaCl, 1 mM CaCl_2_, 1 mM MgCl_2_, which was then mixed with the 120 μL resin. The slurry was then rotated overnight at 4°C to ensure homogeneous distribution of the resin and to allow Fab cleavage by HRV 3C. After overnight cleavage, the slurry was centrifuged and the supernatant containing the ternary complex was collected. This procedure was performed in parallel but with buffer instead of RTX751 in the initial binding reaction for the immunoprecipitation negative control.

#### Cryo-electron microscopy

Immunoprecipitation elution (3 μL) containing α_M_β_2_+RTX751+M1F5 Fab was deposited onto a 1.2/1.3 UltrAuFoil holey gold grid (Electron Microscopy Sciences) that had been plasma cleaned for 4 min using a Gatan Solarus 950 with a 4:1 O2:H2 ratio. A Vitrobot Mark IV was used to plunge-freeze the sample, with a blot force of 1, a blot time of 4s, a humidity level of 100%, and at 4°C. Movies were then collected using a K3 detector at a magnification of 22,500× (corresponding to a pixel size of 1.073 Å) in a Titan Krios operating at 300 kV, using 80 e–/Å2 total dose. Defocus values were varied from −0.8 μm to −2.2 μm. A full description of the data collection parameters can be found in Table S1. Motion correction, CTF estimation, and particle picking were performed in Warp (Tegunov and Cramer, 2019). Particles were subsequently transferred to cryoSPARC v3.2 (Punjani et al., 2017) for 2D classification and 3D reconstruction. The globally refined map as well as the locally refined maps were sharpened using DeepEMhancer (Sanchez-Garcia et al., 2021). Sharpened global and local maps were combined using the Phenix combine_focused_maps function in Phenix (Adams et al., 2002). Model building and refinement were subsequently performed using Coot, Phenix and ISOLDE (Adams et al., 2002; Croll, 2018; Emsley and Cowtan, 2004). Structural biology applications used in this project were compiled and configured by SBGrid (Morin et al., 2013).

### QUANTIFICATION AND STATISTICAL ANALYSIS

No quantification or statistical tests were performed.

## Supplementary Material

1

## Figures and Tables

**KEY RESOURCES TABLE:** 

REAGENT or RESOURCE	SOURCE	IDENTIFIER

Antibodies		

Anti-ACT RTX domain antibody, M1F5	This manuscript	N/A

Bacterial and virus strains		

*E. coli* BL21 (DE3)	New England Biolabs	Cat# C2527H

Chemicals, peptides, and recombinant proteins		

Integrin α_M_β_2_ protein	This manuscript	N/A
Integrin α_X_β_2_ protein	This manuscript	N/A
Integrin α_M_β_2_(αM 597–665) protein	This manuscript	N/A
RTX751 protein	This manuscript	N/A
FreeStyle™ 293 Expression Medium	Gibco	Cat# 12338002
OPTI-MEM, Reduced Serum Medium	ThermoFisher	Cat# 11058021
25 kDa linear polyethylenimine	Polysciences	Cat# 3966-2

Deposited data		

CryoEM structure of α_M_β_2_+RTX751+M1F5 Fab	This manuscript	PDB ID: 7USL
α_M_β_2_+RTX751+M1F5 Fab composite EM map	This manuscript	EMDB ID: 26738
α_M_β_2_+RTX751+M1F5 Fab global refinement EM map	This manuscript	EMDB ID: 27122
α_M_β_2_+RTX751+M1F5 Fab tailpiece local EM map	This manuscript	EMDB ID: 27123
α_M_β_2_+RTX751+M1F5 Fab acylation local EM map	This manuscript	EMDB ID: 27124
Cryo-EM structure of α_M_β_2_	This manuscript	PDB ID: 7USM
α_M_β_2_ composite EM map	This manuscript	EMDB ID: 26739
α_M_β_2_ global refinement EM map	This manuscript	EMDB ID: 27125
α_M_β_2_ tailpiece local refinement EM map	This manuscript	EMDB ID: 27126

Experimental models: Cell lines		

Freestyle 293-F cells	ThermoFisher Scientific	Cat# R79007

Recombinant DNA		

pαH-α_M_	This manuscript	N/A
pαH-α_X_	This manuscript	N/A
pαH-(αM 597–665)	This manuscript	N/A
pαH-β_2_	This manuscript	N/A
pET22b-RTX751	This manuscript	N/A
pVRC8400-M1F5_HC	This manuscript	N/A
pVRC8400-M1F5_LC	This manuscript	N/A

Software and algorithms		

GraphPad Prism	Motulsky and Brown (2006)	V9.0.2
Biacore X100 Evaluation Software	GE Healthcare	V2.0.1
ISOLDE	Croll (2018)	V1.1.0
COOT	Emsley and Cowtan (2004)	http://bernhardcl.github.io/coot/
Phenix	Adams et al. (2002); Afonine et al. (2018)	https://www.phenix-online.org/
ChimeraX	Goddard et al. (2018)	https://www.rbvi.ucsf.edu/chimerax/
cryoSPARC	Punjani et al. (2017)	V2.15.0

Other		

HisPur™ Ni-NTA Resin	ThermoFisher Scientific	Cat# 88223
Pierce™ Protein A Agarose	ThermoFisher Scientific	Cat# 20334
Biacore X100 Sensorchip CM5	Cytiva	Cat# 29149604
EDC/NHS amine coupling kit	Cytiva	Cat# BR100050
Superdex 200 increase 10/300 GL	Cytiva	Cat# 28990944
HiPrep 26/10 desalting	Cytiva	Cat# 17508701
Quantifoil® Holey Gold Film R 1.2/1.3, Gold, 300 mesh	Electron Microscopy Sciences	Cat# Q350AR13A

## Highlights

- Cryo-EM structure of *Bordetella* adenylate cyclase toxin (ACT) bound to integrin α_M_β_2_
- ACT interacts with the α_M_β_2_ headpiece and tailpiece, preferring the bent conformation
- α_M_β_2_ binding positions the ACT acylation sites at the host cell membrane for insertion
- Neutralizing ACT antibodies prevent attachment by sterically clashing with α_M_β_2_
