# Supplementary material for: Structural basis for non-canonical integrin engagement by Bordetella adenylate cyclase toxin
Source: Cell Rep. Author manuscript; Available in PMC 2022 Aug 26. (PMC9416875; doi:10.1016/j.celrep.2022.111196)
Supplement: 1 [file NIHMS1830636-supplement-1.pdf]

Cell Reports, Volume *40*

Supplemental information

Structural basis for non-canonical integrin engagement by *Bordetella* adenylate cyclase toxin

Jory A. Goldsmith, Andrea M. DiVenere, Jennifer A. Maynard, and Jason S. McLellan

Figure S1

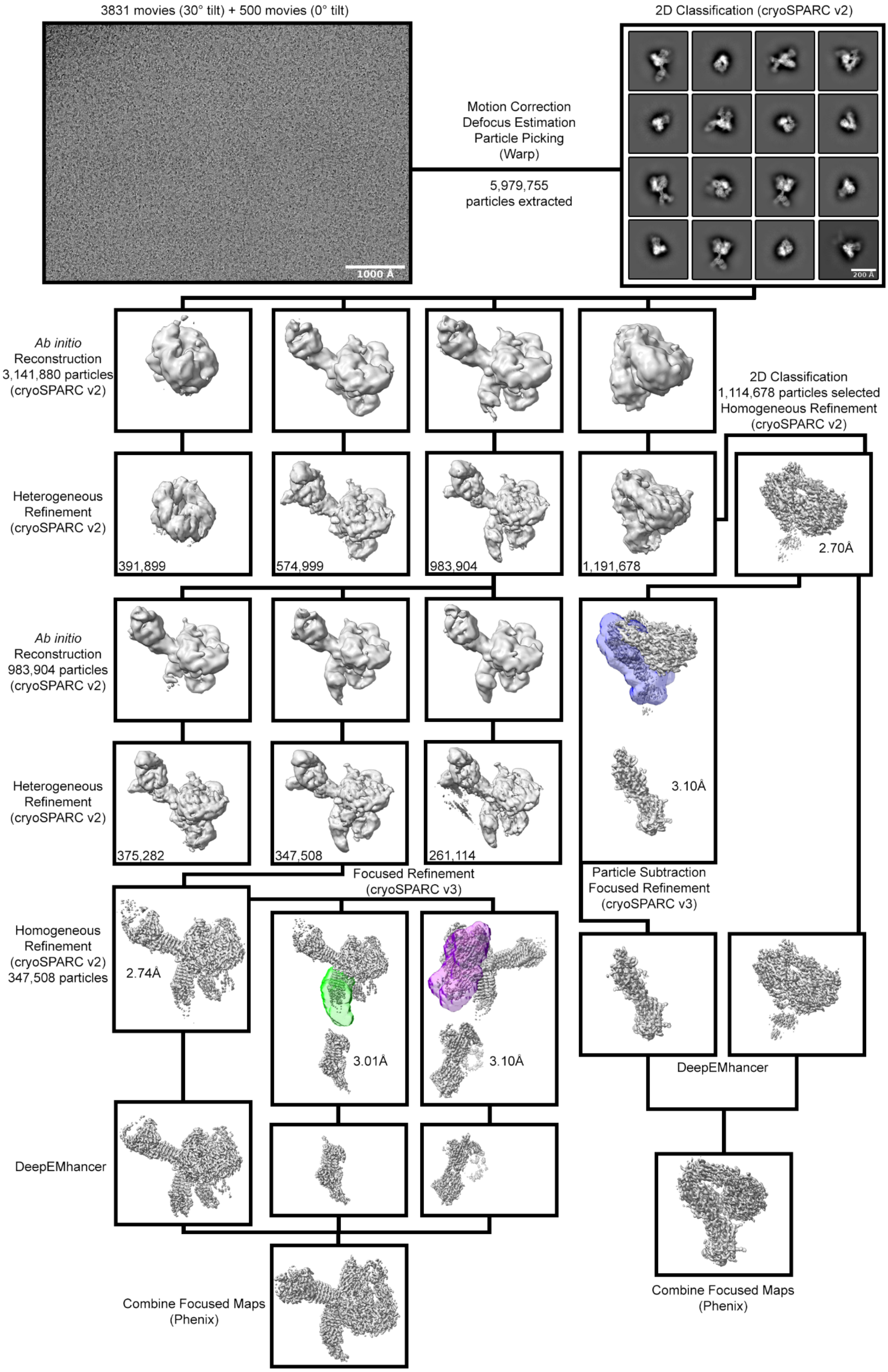

3831 movies (30° tilt) + 500 movies (0° tilt)
1000 Å
2D Classification (cryoSPARC v2)
200 Å
Motion Correction
Defocus Estimation
Particle Picking
(Warp)
5,979,755
particles extracted
Ab initio
Reconstruction
3,141,880 particles
(cryoSPARC v2)
2D Classification
1,114,678 particles selected
Homogeneous Refinement
(cryoSPARC v2)
Heterogeneous
Refinement
(cryoSPARC v2)
391,899
574,999
983,904
1,191,678
2.70Å
Ab initio
Reconstruction
983,904 particles
(cryoSPARC v2)
3.10Å
Heterogeneous
Refinement
(cryoSPARC v2)
375,282
347,508
261,114
Focused Refinement
(cryoSPARC v3)
Particle Subtraction
Focused Refinement
(cryoSPARC v3)
Homogeneous
Refinement
(cryoSPARC v2)
347,508 particles
2.74Å
3.01Å
3.10Å
DeepEMhancer
DeepEMhancer
Combine Focused Maps
(Phenix)
Combine Focused Maps
(Phenix)

Figure S1. Cryo-EM data processing workflow, Related to Figure 2.
The number of particles after 2D classification, as well as after each round of 3D heterogeneous refinement are shown. For local refinements, the corresponding global refinement and transparent mask are shown, with the reconstruction resulting from local refinement shown below the global reconstruction. The acylation domain mask is colored green, the bound α_M tailpiece mask is purple, and the unbound α_M tailpiece mask is blue.

Figure S2

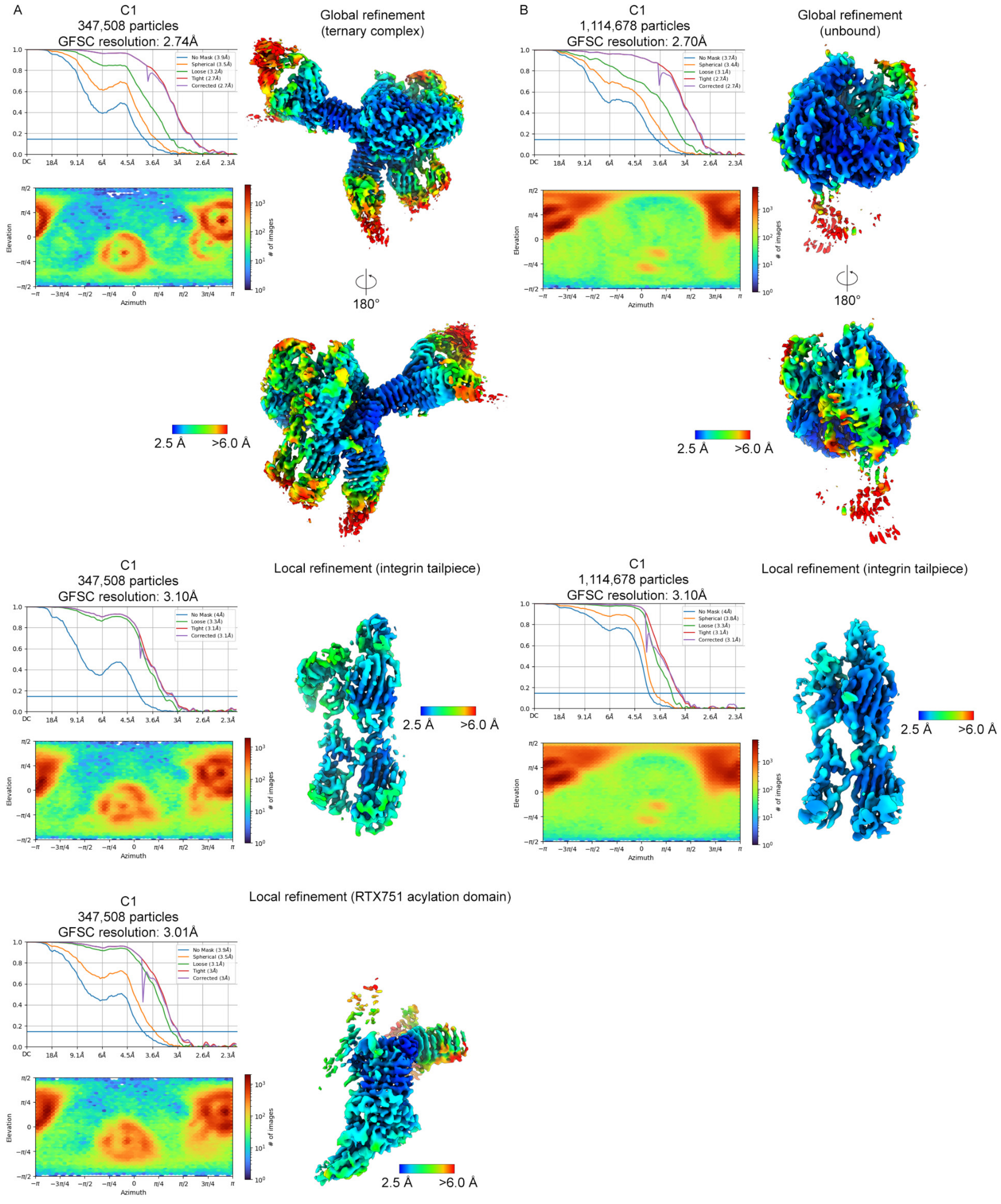
A
C1
347,508 particles
GFSC resolution: 2.74Å
No Mask (3.9Å)
Spherical (3.5Å)
Loose (3.2Å)
Tight (2.7Å)
Corrected (2.7Å)
Global refinement
(ternary complex)
Elevation
Azimuth
# of images
180°
2.5 Å
>6.0 Å
B
C1
1,114,678 particles
GFSC resolution: 2.70Å
No Mask (3.7Å)
Spherical (3.4Å)
Loose (3.1Å)
Tight (2.7Å)
Corrected (2.7Å)
Global refinement
(unbound)
180°
2.5 Å
>6.0 Å
C1
347,508 particles
GFSC resolution: 3.10Å
No Mask (4Å)
Loose (3.3Å)
Tight (3.1Å)
Corrected (3.1Å)
Local refinement (integrin tailpiece)
2.5 Å
>6.0 Å
C1
1,114,678 particles
GFSC resolution: 3.10Å
No Mask (4Å)
Spherical (3.8Å)
Loose (3.3Å)
Tight (3.1Å)
Corrected (3.1Å)
Local refinement (integrin tailpiece)
2.5 Å
>6.0 Å
C1
347,508 particles
GFSC resolution: 3.01Å
No Mask (3.9Å)
Spherical (3.5Å)
Loose (3.1Å)
Tight (3Å)
Corrected (3Å)
Local refinement (RTX751 acylation domain)
2.5 Å
>6.0 Å

Figure S2. Cryo-EM data processing validation, Related to Figure 2.

Fourier shell correlation (FSC) curves used for resolution determination are shown. Below the FSC curves are the view plots showing the particle orientations for each reconstruction. Maps are rainbow-colored based on estimated local resolution, from blue (high) to red (low), with the range covered by the rainbow color palette denoted in the legend.

Figure S3

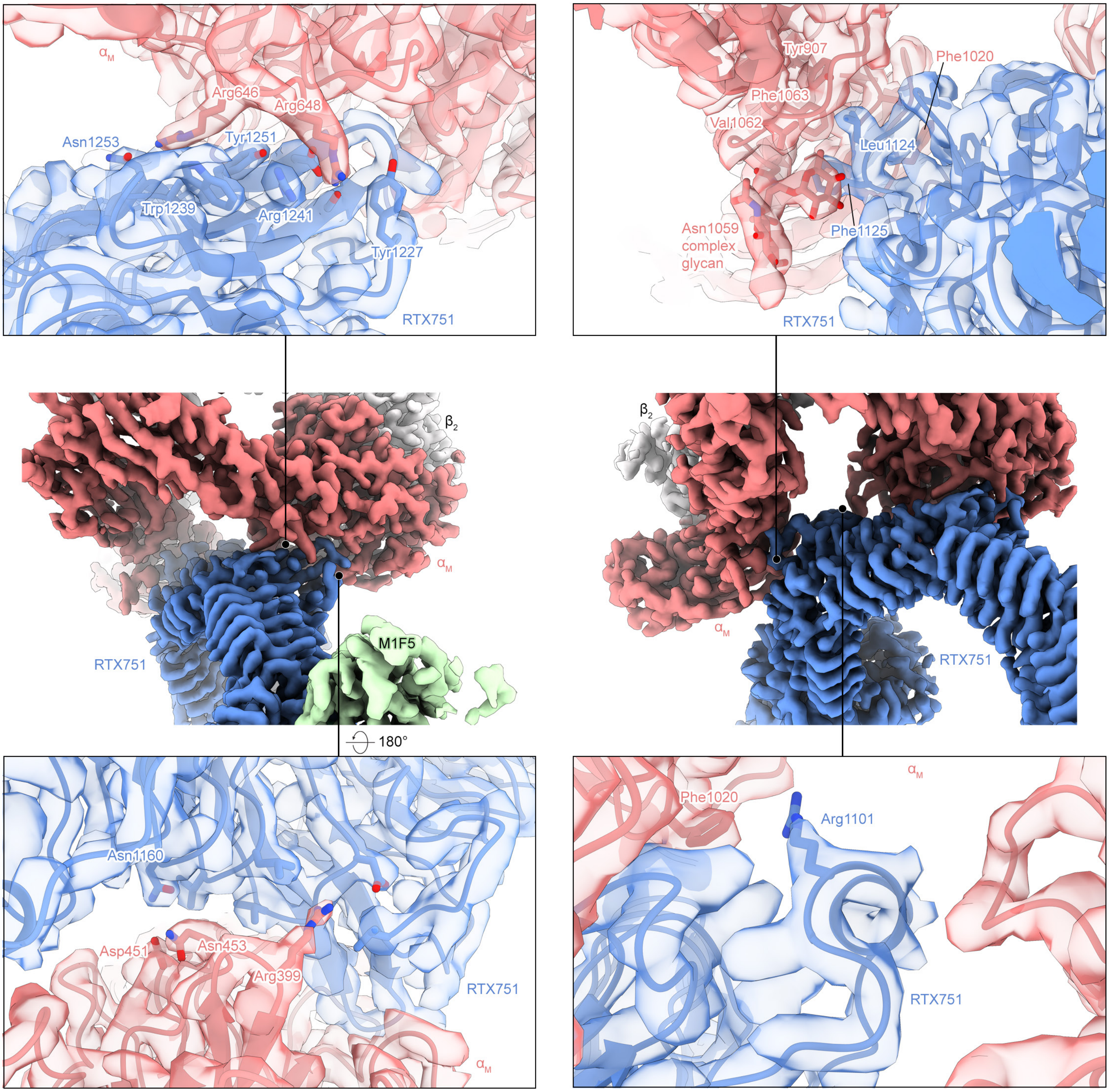

Figure S3. Cryo-EM map at the RTX751-α_M interface, Related to Figure 3.
The cryo-EM reconstruction of the $\alpha_M\beta_2$ ectodomain in complex with RTX751 and M1F5 Fab is shown as an opaque surface. The map is shown with α_M colored pink, β_2 colored white, RTX751 colored blue, M1F5 heavy chain colored green, and M1F5 light chain colored pale green. Insets show transparent cryo-EM maps and ribbon representation of built models, with stick representation of key interface residues.

Figure S4

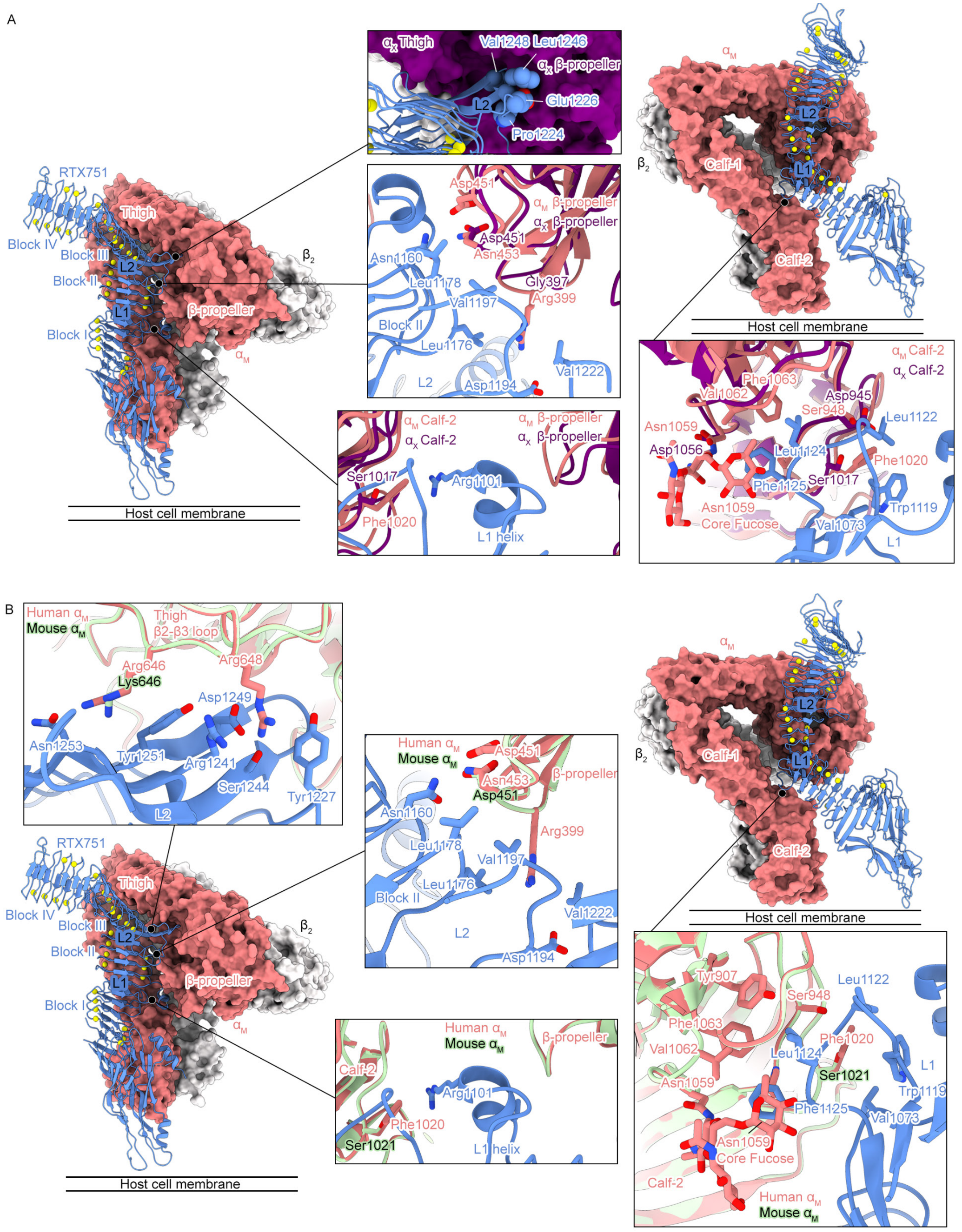
A
α_X Thigh
Val1248 Leu1246
α_X β-propeller
L2
Glu1226
Pro1224
α_M
L2
L1
β_2
Calf-1
Calf-2
Host cell membrane
RTX751
Thigh
Block IV
Block III
L2
Block II
L1
Block I
β-propeller
β_2
α_M
Host cell membrane
Asp451
α_M β-propeller
α_X β-propeller
Asp451
Asn453
Asn1160
Leu1178
Gly397
Arg399
Val1197
Block II
Leu1176
L2
Asp1194
Val1222
α_M Calf-2
α_X Calf-2
α_M β-propeller
α_X β-propeller
Ser1017
Arg1101
Phe1020
L1 helix
α_M Calf-2
α_X Calf-2
Phe1063
Val1062
Asp945
Ser948
Leu1122
Asn1059
Asp1056
Leu1124
Phe1020
Phe1125
Ser1017
Asn1059
Core Fucose
Trp1119
Val1073
L1
B
Human α_M
Mouse α_M
Thigh
β2-β3 loop
Arg646
Lys646
Arg648
Asp1249
Asn1253
Tyr1251
Arg1241
Ser1244
Tyr1227
L2
RTX751
Thigh
Block IV
Block III
L2
Block II
L1
Block I
β-propeller
β_2
α_M
Host cell membrane
Human α_M
Mouse α_M
Asp451
Asn453
β-propeller
Asp451
Asn1160
Arg399
Leu1178
Val1197
Leu1176
Block II
Val1222
L2
Asp1194
Human α_M
Mouse α_M
β-propeller
Calf-2
Arg1101
Phe1020
Ser1021
L1 helix
α_M
L2
L1
β_2
Calf-1
Calf-2
Host cell membrane
Tyr907
Ser948
Leu1122
Phe1063
Val1062
Phe1020
Leu1124
L1
Ser1021
Asn1059
Trp1119
Phe1125
Val1073
Asn1059
Core Fucose
Calf-2
Human α_M
Mouse α_M

Figure S4. Conservation of ACT-binding α_M residues in α_X and mouse α_M, Related to Figure 3.

(A) Model of the RTX751–$\alpha_M\beta_2$ complex, with regions shown in the insets denoted by black circles. Insets show α_X superimposed with α_M at the ACT-binding interface. Interface residues of α_M and RTX751 are shown as sticks or spheres and are labelled. α_X residues that differ from α_M interface residues are shown as sticks and are labeled. To superimpose α_X with the RTX751-$\alpha_M\beta_2$ model, the β-propeller, thigh, and calf-2 domains were aligned individually [PDB ID: 3K6S (Xie et al., 2010)] to the corresponding α_M domain within the ternary complex. α_X is colored purple, RTX751 is colored blue, α_M is colored pink, and β_2 is colored white. Oxygen atoms are red, nitrogens are blue, and calcium ions are shown as yellow spheres. (B) Model of the RTX751–$\alpha_M\beta_2$ complex, with the regions shown in the insets denoted by black circles. Insets show a homology model of mouse α_M superimposed with human α_M at the ACT-binding interface. Interface residues of human α_M and RTX751 are shown as sticks and are labelled. Mouse α_M residues that differ from human α_M interface residues are shown as sticks and are labeled. The homology model of mouse α_M was generated using SWISS-MODEL(Waterhouse et al., 2018), and the β-propeller, thigh, and calf-2 of the homology model were aligned as single domains to human α_M from the ternary complex. Mouse α_M is colored light green, and the remaining coloring is the same as in (A).

Figure S5

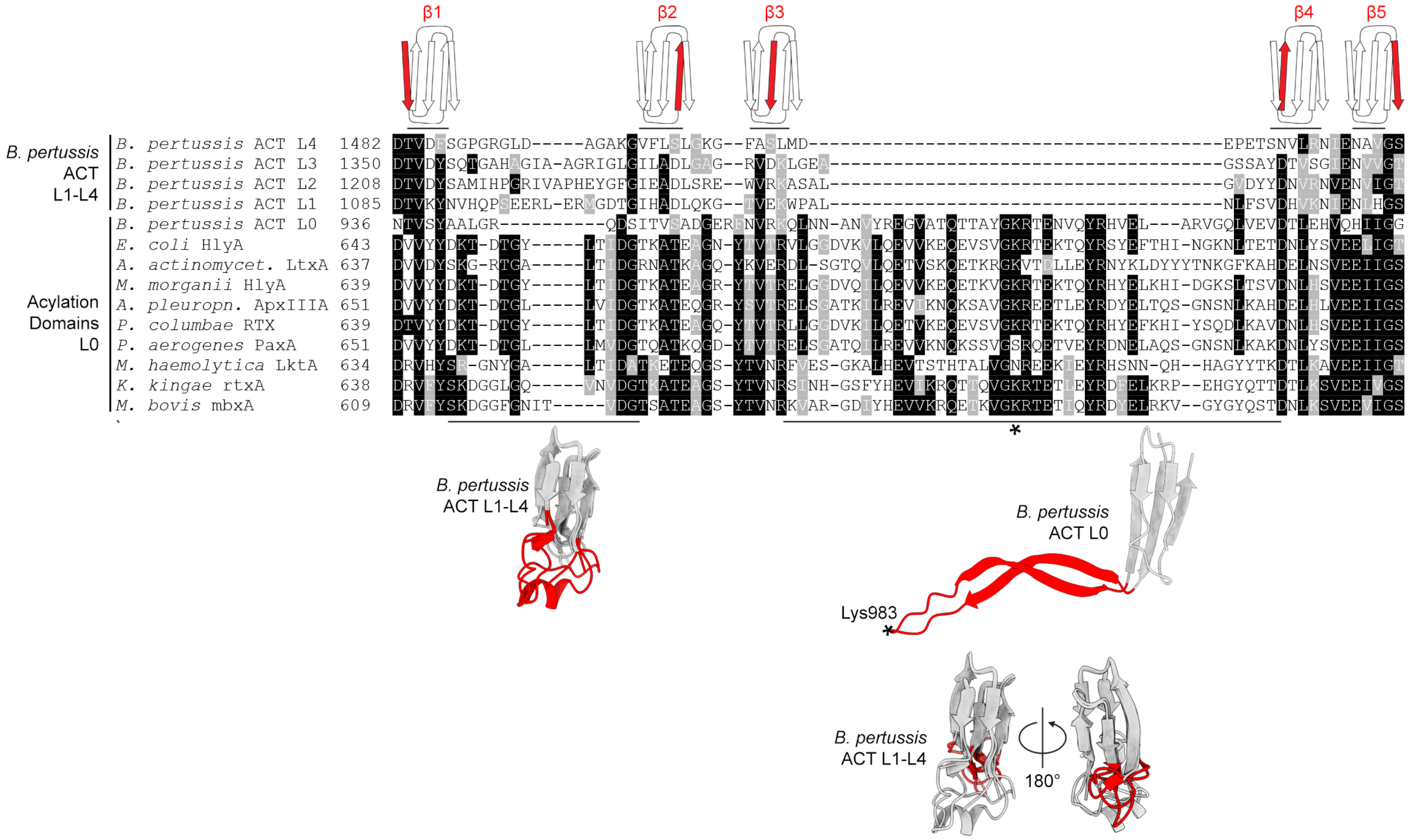

Figure S5. Amino acid sequence alignment of the conserved linker motifs of *B. pertussis* ACT and other pore-forming RTX toxins, Related to Figure 5.

Aligned sequence depicting the conservation between *B. pertussis* ACT RTX domain linkers 1–4, *B. pertussis* ACT linker 0 from the acylation domain, and linker 0 from the conserved acylation domain of other pore-forming RTX toxins. The regions of the alignment corresponding to the core β-sheets of the linker motif are denoted by a horizontal line, with a topology diagram of the motif above the line denoting the corresponding β-sheet colored red. For both the β1-β2 and β3-β4 loops, a structural alignment of *B. pertussis* ACT L1–L4 (L4 from PDB ID: 6SUS) is shown using ribbon representation, with the corresponding loops colored red and the remaining portions of the linkers colored white. For the β3-β4 loop, *B. pertussis* L0 is shown in ribbon representation. The β3-β4 loop of L0, which contains the Lys983 acylation site, is colored red, and the remaining portions of L0 are colored white.

Figure S6

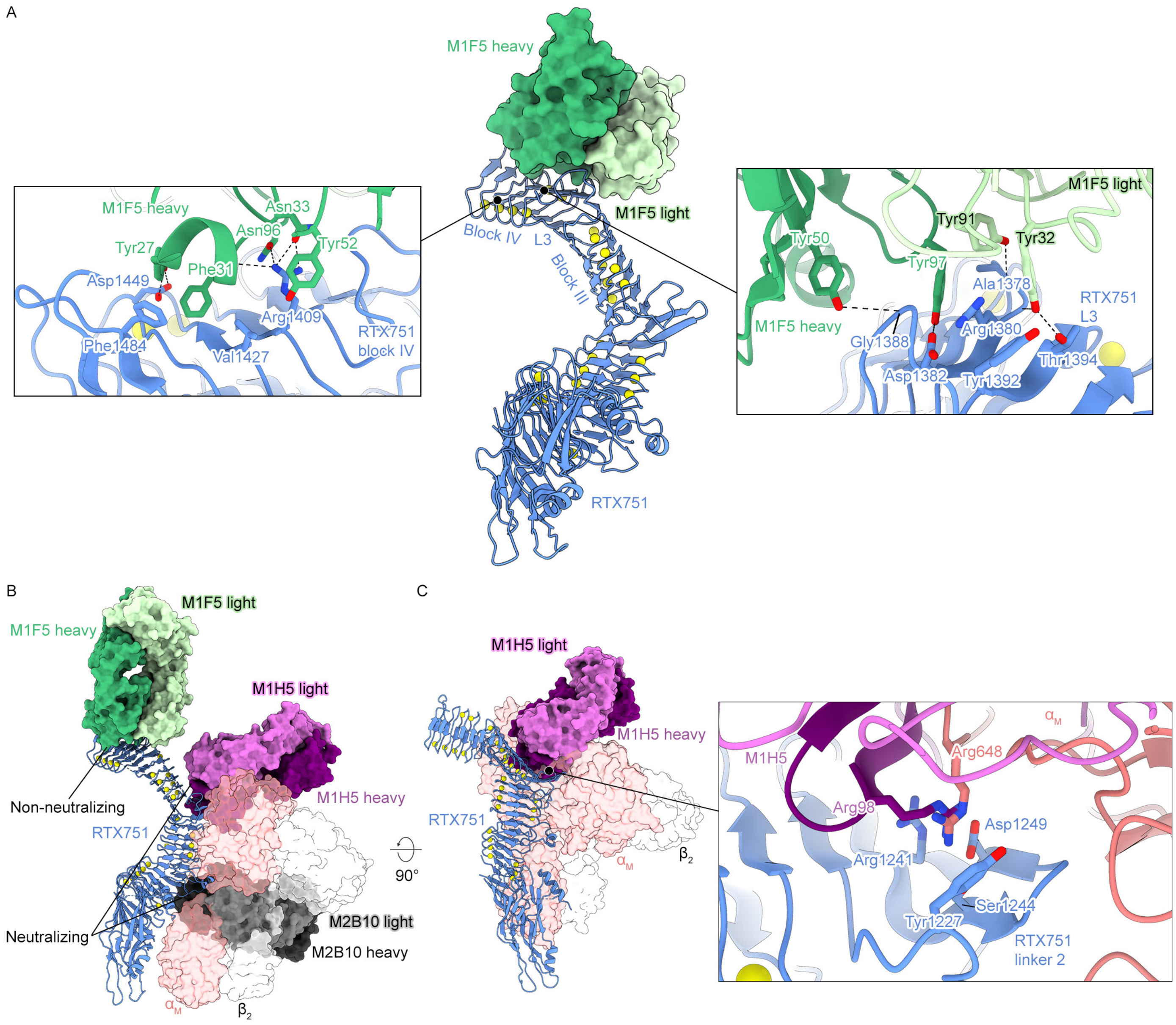
A
M1F5 heavy
M1F5 light
Block IV
L3
Block III
RTX751
M1F5 heavy
Asn33
Asn96
Tyr27
Tyr52
Phe31
Asp1449
Arg1409
RTX751
block IV
Phe1484
Val1427
M1F5 light
Tyr91
Tyr50
Tyr32
Tyr97
Ala1378
RTX751
L3
M1F5 heavy
Arg1380
Gly1388
Thr1394
Asp1382
Tyr1392
B
M1F5 light
M1F5 heavy
M1H5 light
M1H5 heavy
Non-neutralizing
RTX751
90°
Neutralizing
M2B10 light
M2B10 heavy
αM
β2
C
M1H5 light
M1H5 heavy
RTX751
αM
β2
αM
M1H5
Arg648
Arg98
Asp1249
Arg1241
Ser1244
Tyr1227
RTX751
linker 2

Figure S6. The RTX linkers of ACT are immunological hot spots. Related to Figure 3. (A) The model of RTX751 bound to M1F5 Fab is shown. RTX751 is colored blue, M1F5 Fab heavy chain is colored green, and M1F5 Fab light chain is colored pale green. RTX751 is shown in ribbon representation with calcium ions shown as yellow spheres, and M1F5 Fab is shown as a molecular surface. Black circles denote the locations of the interface regions shown in the insets. Key interface residues shown as sticks. (B) Composite model containing $\alpha_M\beta_2$+RTX751+M1F5 Fab with M2B10 Fab and M1H5 Fab superimposed based on a crystal structure of these antibodies in complex with an RTX domain fragment [PDB ID: 7RAH (Goldsmith et al., 2021)]. The modelled Fabs are shown using surface representation. M1F5 is colored green/pale green, M1H5 is colored purple/pink, and M2B10 is colored charcoal/grey. $\alpha_M\beta_2$ is shown as a semi-transparent molecular surface with α_M colored pink and β_2 colored white. RTX751 is shown in ribbon representation and colored blue. Calcium ions are represented by yellow spheres. (C) The model of the RTX751–$\alpha_M\beta_2$ complex is shown with M1H5 Fab superimposed. The region shown in the inset is denoted by a black circle. The inset shows the interactions formed by M1H5 Arg98 with the ACT RTX domain superimposed onto the interactions formed by α_M Arg648 with the ACT RTX domain. RTX751, α_M, and M1H5 Fab are shown as ribbons with key residues shown as sticks.

Table S1. Cryo-EM data collection and refinement statistics. Related to Figure 2.

**EM data collection**	
Microscope	FEI Titan Krios
Detector	Gatan K3
Defocus range (μm)	0.8-2.2
Pixel size (Å)	1.073
Magnification (nominal)	22,500
Voltage (kV)	300
Exposure rate (e^-/pix/sec)	8
Frames per exposure	80
Micrographs collected	3831 (30˚tilt)<br>500 (0˚tilt)
Micrographs used	3429 (30˚tilt)<br>432 (0˚tilt)
Electron exposure (e^-/Å^2)	80
Particles extracted (total)	5,979,755
Automation software	SerialEM

**3D reconstruction statistics**	**$\alpha_M\beta_2$+RTX751+M1F5**			**$\alpha_M\beta_2$**	
Final particles	347,508			1,114,678	
Reconstruction	Global	$\alpha_M\beta_2$ tailpiece	Acylation domain	Global	$\alpha_M\beta_2$ tailpiece
Symmetry	C1	C1	C1	C1	C1
Unmasked resolution at 0.5 FSC (Å)	5.3	5.7	5.4	5.1	4.6
Masked resolution at 0.5 FSC (Å)	3.5	4.0	3.8	3.5	3.9
Unmasked resolution at 0.143 FSC (Å)	3.9	4.0	3.9	3.7	4.0
Masked resolution at 0.143 FSC (Å)	2.7	3.1	3.0	2.7	3.1

Table S1. Cryo-EM data collection and refinement statistics (continued).

**Model refinement and validation**	**$\alpha_M\beta_2$+RTX751+M1F5**	**$\alpha_M\beta_2$**
Refinement package	Phenix	
Refinement tool	Real-space refinement	
Refinement strategies	min global, local_grid_search, adp, ss restraints, rotamer restraints, Ramachandran restraints	
Initial model(s) used (PDB ID)	3K6S, 7RAH	3K6S
<u>Model composition</u>		
Number of Atoms	19042	11905
Protein	18791	11705
N-glycan	218	196
Ca^{2+}	33	4
Protein B-factors (mean)	61.2	57.6
RMSD bond lengths (Å)	0.003	0.003
RMSD bond angles (°)	0.58	0.60
MolProbity score	1.65	1.89
Clashscore	5.3	8.2
Rotamer outliers (%)	0.0	0.1
C-beta outliers (%)	0.0	0.0
Ramachandran plot		
Favored (%)	96.8	97.0
Allowed (%)	3.2	3.0
Outliers (%)	0.0	0.0
EMRinger score	3.65	2.63
CaBLAM outliers (%)	2.9	2.3
CC (mask)	0.69	0.68

**Data Availability**		
EMDB	EMD-26738 (composite)	EMD-26739 (composite)
	EMD-27122 (global)	EMD-27125 (global)
	EMD-27123 (tailpiece)	EMD-27126 (tailpiece)
	EMD-27124 (acylation)	
